# The importance of mechanical conditions in the testing of excitation abnormalities in a population of electro-mechanical models of human ventricular cardiomyocytes

**DOI:** 10.3389/fphys.2023.1187956

**Published:** 2023-06-08

**Authors:** Arsenii Dokuchaev, Alexander Kursanov, Nathalie A. Balakina-Vikulova, Leonid B. Katsnelson, Olga Solovyova

**Affiliations:** ^1^ Laboratory of Mathematical Physiology, Institute of Immunology and Physiology, Ural Branch of Russian Academy of Sciences, Ekaterinburg, Russia; ^2^ Laboratory of Mathematical Modeling in Physiology and Medicine Based on Supercomputers, Ural Federal University, Ekaterinburg, Russia

**Keywords:** mathematical models, human ventricular cardiomyocyte, mechanical function, cardiac electrophysiology, repolarization abnormalities, drug testing

## Abstract

**Background:** Populations of *in silico* electrophysiological models of human cardiomyocytes represent natural variability in cell activity and are thoroughly calibrated and validated using experimental data from the human heart. The models have been shown to predict the effects of drugs and their pro-arrhythmic risks. However, excitation and contraction are known to be tightly coupled in the myocardium, with mechanical loads and stretching affecting both mechanics and excitation through mechanisms of mechano-calcium-electrical feedback. However, these couplings are not currently a focus of populations of cell models.

**Aim:** We investigated the role of cardiomyocyte mechanical activity under different mechanical conditions in the generation, calibration, and validation of a population of electro-mechanical models of human cardiomyocytes.

**Methods:** To generate a population, we assumed 11 input parameters of ionic currents and calcium dynamics in our recently developed TP + M model as varying within a wide range. A History matching algorithm was used to generate a non-implausible parameter space by calibrating the action potential and calcium transient biomarkers against experimental data and rejecting models with excitation abnormalities. The population was further calibrated using experimental data on human myocardial force characteristics and mechanical tests involving variations in preload and afterload. Models that passed the mechanical tests were validated with additional experimental data, including the effects of drugs with high or low pro-arrhythmic risk.

**Results:** More than 10% of the models calibrated on electrophysiological data failed mechanical tests and were rejected from the population due to excitation abnormalities at reduced preload or afterload for cell contraction. The final population of accepted models yielded action potential, calcium transient, and force/shortening outputs consistent with experimental data. In agreement with experimental and clinical data, the models demonstrated a high frequency of excitation abnormalities in simulations of Dofetilide action on the ionic currents, in contrast to Verapamil. However, Verapamil showed a high frequency of failed contractions at high concentrations.

**Conclusion:** Our results highlight the importance of considering mechanoelectric coupling *in silico* cardiomyocyte models. Mechanical tests allow a more thorough assessment of the effects of interventions on cardiac function, including drug testing.

## 1 Introduction

The use of populations of *in silico* cardiac models is actively applied in the simulation of physiological objects and phenomena in the heart. This approach employs many different sets of input parameters for the mathematical description of an object rather than just one. The aggregation of input parameter sets and mathematical description of an object provide a population of mathematical models. The population of models can then be used to analyze and predict variability in the responses of the natural organ/cell/tissue population to various types of exposure. By analogy with the analysis of a set of experimental recordings, a statistical analysis of the entire set of model responses in the population provides information about the statistically significant influence of the test factors on the characteristics of the processes being studied.

Populations of models have been effectively used to study the function of the heart (Ni et al., 2018). The team of Prof. B. Rodriguez has developed populations of electrophysiological models of ventricular cardiomyocytes in animals (Gemmell et al., 2016) and human (Muszkiewicz et al., 2016; Passini et al., 2020). The models in the populations are selected so that the output parameters of the models fall within the range of acceptable values that can be estimated from experimental data. In the research work of this team, priority is given to studying arrhythmia conditions and the cardiotoxicity of various pharmacological compounds (Passini et al., 2019; Tomek et al., 2019; Paci et al., 2020; Margara et al., 2021). There is another approach to assessing uncertainty in model predictions. Pathmanathan and Gray built a population of the canine action potential (AP) models on the basis of a complete set of input parameters whose ranges were determined experimentally (Pathmanathan et al., 2020). Estimating the variability of the model parameters allowed them to determine the effects of parameter uncertainties on the prediction of AP characteristics under drug testing and on the dynamics of spiral waves.

The distinctive feature of our study is the creation of a population of cellular models based on a mathematical description of the human ventricular cardiomyocyte, which combines both the electrophysiology of the cell (AP and ionic currents, intracellular calcium dynamics) and the mechanical function of the cardiomyocyte (force generation and length changes). Such a description allows us to obtain a physiologically consistent population of mathematical models of human ventricular cardiomyocytes calibrated and validated by both the experimental properties of the AP and the time and amplitude characteristics of the force, as well as the characteristic dependencies of mechanical variables (e.g., ‘length - force’, *etc.*) obtained in the experiment.

## 2 Materials and methods

### 2.1 Electro-mechanical model of the human ventricular cardiomyocyte (TP + M model)

In this research work, we use the mathematical description of the electro-mechanical activity of human ventricular cardiomyocytes that we developed recently (Balakina-Vikulova et al., 2020). Here, we utilize an improved variant of the model with a corresponding set of input model parameters as recently described in (Bazhutina et al., 2021). Hereafter in the text, this model is reffered to as a reference TP + M model, and we use the latter parameter set as a starting point to build a population of models.

The TP + M model combines the description of cardiomyocyte electrophysiology from the ten Tusscher-Panfilov (TP) model (ten Tusscher and Panfilov, 2006) with the original description of the mechanical (M) activity of the cardiac muscle developed by the Ekaterinburg team (Sulman et al., 2008). It contains a biophysically detailed description of ion channels, pumps, and exchange currents, as well as a detailed description of intracellular sodium (*Na* ^+^), calcium (*Ca* ^2+^), and potassium (*K* ^+^) kinetics. The description of intracellular *Ca* ^2+^ kinetics interlinks both the electrical and mechanical blocks of the model. The description of the *Ca* ^2+^ release via the ryanodine receptor channels was improved in the TP + M model compared to the original TP model (Bazhutina et al., 2021).

The mechanical activity of the virtual cardiomyocyte is described by the rheological scheme (Figure 2 in Balakina-Vikulova et al., (2020)), which contains a contractile element representing the sarcomeres and elastic and viscous elements that can be used to describe the passive and viscous properties of the cardiac preparation. The mechanical part of the model describes the time dependent deformations and the force generation in the elements of the rheological scheme during contraction cycle. The equations for the contractile element describe the generation of active force in the cardiomyocytes as a result of cross-bridge (Xb) formation by myosin heads attaching to the thin actin filaments.

Interactions between the electrical and mechanical activity in the cardiomyocytes realized through the mechanisms of the excitation-contraction coupling and mechano-electric feedback (mechano-electric coupling) (Quinn and Kohl, 2021). There are two main mechanisms of mechano-electric coupling that are generally considered in the heart: ionic currents via mechano-sensitive (e.g., stretch-activated) channels, and mechano-dependence of intracellular calcium handling. In our study, we consider only the mechano-calcium-electric feedbacks given by the cooperative mechanisms of the kinetics of calcium-troponin C complex (CaTnC) (Sulman et al., 2008). We allow for the slowing down of CaTnC decay when a larger number of Xbs and/or a larger number of other CaTnC form in its vicinity along the actin filament. Dependence of the Xb attachment/detachment on sarcomere length also affects CaTnC kinetics via cooperative effects of a bound Xb on the affinity of CaTnC complexes.

The TP + M model was validated using experimental data from human cardiac muscle preparations. It reproduced well the main temporal and amplitude characteristics of AP, *Ca* ^2+^ transient (time-dependent change in the concentration of free *Ca* ^2+^ in the cytosol) and force twitches (time-dependent change in the generated force) during excitation-contraction cycle under isometric conditions at different mechanical preloads (initial cell lengths) and contractions at different afterloads applied to the virtual cardiomyocyte (Balakina-Vikulova et al., 2020; Bazhutina et al., 2021).

Then, we additionally validated the TP + M model with the experimental data summarized in (Margara et al., 2021). Some of these data were then used here as biomarkers of the electrical and mechanical function of cardiomyocytes when building a population of models (Table 1). Some other data served to validate and evaluate the resulting population of calibrated electro-mechanical models (see Results section).

**TABLE 1:** Experimental biomarkers used for calibrating and evaluating the population based on the TP + M model. *RMP* – resting potential; *APD*_*xx*_ – AP duration at XX% of repolarization; *Tri*_9040_ – AP triangulation, defined as the difference between *APD*_90_ and *APD*_40_; *CT*_min_, *CT*_max_ – *Ca* ^2+^ transient diastolic and peak values; *CaTTP* – *Ca* ^2+^ transient time to peak; *CTD*_*xx*_ – *Ca* ^2+^ transient duration at XX% of decay; *FTTP* – time to isometric peak force; *FTTr* – time to 90% decay of isometric force from peak value; *FTD* – isometric twitch duration. *n*/*a* is for the biomarkers whose values were not described in the corresponding experimental papers, and which could not be determined on the basis of other biomarkers from these experiments.

Calibration			
Data type	Biomarkers (min, mean, max; *σ*)	Tissue preparation	References
Rest membrane potential, mV	*RMP* (−100, −86, −70; n/a)	M-cells from the left ventricular wall of human heart	Drouin et al. (1995)
Action potential duration, ms	*APD*_20_ (86, 172, 262; 29)	Human ventricular trabeculae from normal donor heart (paced at 1 Hz, 37 °C)	Page et al. (2016)
	*APD*_50_ (117, 230, 351; 39)		
	*APD*_90_ (170, 311, 464; 49)		
	*Tri*_9040_ (27, 135, 261; 39)		
Diastolic *Ca* ^2+^ concentration, *μ*M	*CT*_min_ (n/a, 0.152, 0.242; 0.03)	Left ventricular trabeculae from non-failing hearts (paced at 1 Hz, 37 °C)	Vahl et al. (1998)
Peak *Ca* ^2+^ transient, *μ*M	*CT*_max_ (0.736, 1.120, 1.504; 0.128)	Left ventricular trabeculae from non-failing hearts (paced at 1 Hz, 37 °C)	Vahl et al. (1998)
*Ca* ^2+^ transient time to peak, ms	*CaTTP* (30, n/a, 300; n/a)	Human ventricular trabeculae from organ donors without cardiac disease	Gwathmey et al. (1990)
		Adult human primary ventricular myocytes isolated from donor hearts	Piacentino et al. (2003)
*Ca* ^2+^ transient duration, ms	*CTD*_50_ (55, n/a, 270; n/a)	Ventricular muscle strip preparations from nonfailing human hearts (paced at 1 Hz, 37 °C)	Pieske et al. (1995)
	*CTD*_80_ (120, n/a, 465; n/a)	Isolated left ventricular wedge preparations from nonfailing human hearts	Lou et al. (2011)
		Nontransplanted donor hearts	Coppini et al. (2013)
Re-calibration			
Active tension, time to peak, ms	*FTTP* (212, 275, 338; 21)	Single cardiomyocytes of human nonfailing hearts	Brixius et al. (2001)
Active tension, time to 90% decay, ms	*FTTr* (127, 301, 475; 58)	Left ventricular trabeculae from non-failing hearts (paced at 1 Hz, 37 °C)	Vahl et al. (1998)
Active tension, twitch duration, ms	*FTD* (375, 501, 627; 42)	Left ventricular trabeculae from non-failing hearts	Pieske et al. (1996)
Length-Force dependence	Length-dependence of developed force	Isolated intact human left ventricular myocardium from normal donor hearts (paced at 1 Hz, 37 °C)	Vahl et al. (1997)
Length-Force dependence	Length-dependence of developed force	Human left ventricular non-failing preparations from donor hearts (paced at 0.5 Hz, 37 °C)	Holubarsch et al. (1998)
Drug testing			
Action potential duration, ms	*APD*_90_	Adult human primary ventricular myocytes	Nguyen et al. (2017)
Shortening	Sarcomere shortening	Human ventricular trabeculae	Page et al. (2016)

The TP + M model contains 26 nonlinear ordinary differential equations, an additional set of algebraic equations, and a set of input parameters and initial conditions (see Supplementary Materials in (Bazhutina et al., 2021)). The steady-state periodic solution of the system at a stimulation frequency of 1 Hz under isometric conditions at a fixed cell length of 0.93*L*_max_ (where *L*_max_ corresponds to a sarcomere length of 2.23 µm) was employed as a reference model output for comparison with simulations at varying parameters.

Here, we used the TP + M model to create a physiologically plausible population of cellular models by varying the parameters of the TP + M model and obtaining steady-state periodic solutions at 1 Hz stimulation for each set of parameters. A scheme of model selection for a non-implausible population of virtual cardiomyocytes is demonstrated in Supplementary Figure S1 in Supplementary Materials. The first step involves construction of an initial model population calibrated against available experimental data on AP features and *Ca* ^2+^ transient characteristics using the History matching approach (see a subsection below). Then each model from the initial model population is additionally validated in benchmark mechanical simulations to reject models that fail to pass mechanical tests and demonstrate abnormalities in the electrical and/or mechanical activity under different mechanical conditions. The final model population passed the mechanical intervention tests is then validated against the effects of pharmacological substances with experimentally proven properties.

### 2.2 Experimental biomarkers

Experimental studies on the electro-mechanical coupling in the human myocardium are limited due to technical and ethics constraints. Differences in experimental protocols (temperature, stimulation frequency, *Ca* ^2+^-dependent and voltage-dependent dyes, mechanical conditions, washing solution, recording equipment, *etc.*) make the results of different groups hard to compare. Concerning experimental data on the biomarkers used for model calibration, we chose to select data recorded at the stimulation frequency of 1 Hz and temperature of 37°C where available.

Table 1 contains the statistics of the distributions (where available) and the limiting value ranges for AP biomarkers and *Ca* ^2+^ transient characteristics derived from experimental data. These data were used for model calibration during selection.

The use of different *Ca* ^2+^ buffering dyes in the experiments, did not allow us to estimate precisely the duration of the *Ca* ^2+^ transient, so we utilized the maximum and minimum values found in different experiments as cutoff values for permissive models. This may be a rather weak assumption, enlarging the set of non-implausible parameters.

Furthermore, we used experimental biomarkers for contraction only to recalibrate the models, as qualitative experimental data available on force generation in human cardiomyocytes are extremely sporadic (Table 1) (see a subsection below).

### 2.3 History matching approach

An approach called History matching (HM) was developed to solve parameter identification problems and has been used in models of galaxy formation (Vernon et al., 2010), disease epidemiology (Andrianakis et al., 2015), plant physiology (Vernon et al., 2018) and calibration of cardiac cell (Coveney and Clayton, 2018) and anatomical models (Rodero et al., 2023).

In History matching (Supplementary Figure S1), in addition to computational models (called simulators) simulating cardiomyocyte activity for a number of various parameter sets (multi-dimensional vectors or points) from the input parameter space, the approach suggests the use of high-speed regression models (emulators) based, for example, on Gaussian processes (Rasmusen and Williams, 2006). Each emulator predicts a single biomarker value (e.g., action potential duration (APD)), and is trained on the dataset of simulated features derived from the simulator outputs computed at training points from the input space. Such emulators are able to predict the simulated biomarkers for any parameter vector selected from the input parameter space. Emulators take much less time to compute model outputs (typically they are more than 10^6^ times faster than simulators), which makes it possible to quickly estimate the space of input parameters and create a so-called value surface for a specific biomarker. The emulators’ predictions are compared with the biomarker values obtained in the experiments. If the emulator predictions on certain parameter vectors do not meet the calibration criteria, the implausible points are removed from the parameter space.

An input parameter space to develop a population of models was formed of the following 11 input parameters, which values ranged from 0% to 200% of the reference value given in the TP + M model: the conductances of the main transmembrane ionic currents, namely, fast *Na* ^+^ current (*g*_*Na*_), rapid and slow time-dependent *K* ^+^ current (*g*_*Kr*_ and *g*_*Ks*_), inward rectifier *K* ^+^ current (*g*_*K*1_), L-type *Ca* ^2+^ current (*g*_*CaL*_); maximum *Na* ^+^-*K* ^+^ ATPase (*NKX*) current (*P*_*NaK*_) and maximum *Na* ^+^-*Ca* ^2+^ exchanger (*NCX*) current (*K*_*NaCa*_). In addition, we tested several key parameters of *Ca* ^2+^ handling in cells: the maximal velocity of the sarcoplasmic reticulum (SR) ATPase (*V*_*maxup*_), the maximal velocity of SR *Ca* ^2+^ release (*k*_*s*_), and two rate constants in the Markov state ryanodine receptor model (*k*_*im*_, *k*_*om*_).

#### 2.3.1 Output biomarkers

To select physiologically acceptable models, we used biomarkers that characterize the time course of generation of AP and *Ca* ^2+^ transient observed in experimental studies during the contractile cycle of a human cardiac myocyte (Table 1). The following characteristics of the AP and *Ca* ^2+^ transients derived from simulators or predicted by emulators we used to calibrate the models against experimental data (Table 1): resting potential (*RMP*); AP duration at 20%, 50%, 90% of repolarization (*APD*_20_, *APD*_50_, *APD*_90_); AP triangulation (*Tri*_9040_), defined as the difference between *APD*_90_ and *APD*_40_; *Ca* ^2+^ transient time to peak (*CT*_min_); *Ca* ^2+^ transient duration at 50% and 80% of decay (*CTD*_50_, *CTD*_80_).

History matching works iteratively as a series of waves (Supplementary Figure S1) including the following algorithm steps.

#### 2.3.2 Parameter sampling

In the first wave, the input parameters were selected by Latin Hypercube Sampling, which provides good coverage of the entire input space. We sampled 300 sets of input parameters (points) from the 11-dimensional parameter space, where each parameter was sampled from an interval from 0% to 200% of the reference value given in the TP + M model. For subsequent waves, the input parameters were sampled from “Not-ruled-out-yet” (NROY) region of the input space, restricted by the distribution of accepted parameters after filtering emulator outputs.

#### 2.3.3 Simulator calculation

For each input parameter vector, the simulator was computed during 200 cycles at 1 Hz pacing rate to achieve steady-state contractions. The models which demonstrate abnormalities in the time course of AP generation (early or late afterdepolarization, alterations in APD, spontaneous AP, *etc*.), and/or disturbances in the *Ca* ^2+^ transient during the last 2 cycles were rejected.

#### 2.3.4 Emulator training

A set of accepted model parameters and corresponding values of each simulated biomarker (e.g., APD, *Ca* ^2+^ transient amplitude, *etc*.) derived from simulator outputs formed the dataset used to train an emulator based on Gaussian process regression with a radial basis function kernel. Emulator training was performed using simulator data from up to four preceding waves.

#### 2.3.5 Emulator calculation

The next step of the algorithm is to augment the simulated feature dataset by computing the trained emulators for a large number of points from the current NROY parameter space. For the first wave, the emulators were computed for 10^6^ parameter vectors sampled from the input parameter space. For each subsequent wave, the emulators were run for all parameter vectors accepted on the previous wave.

#### 2.3.6 Model calibration

In this algorithm step, the simulator and emulator results (output features) were filtered (calibrated) based on experimental observations, taking into account the variance (uncertainty) of the simulated data and the experimental variability of the biomarker. For each AP biomarker (Table 1), an implausibility measure was calculated as follows (Vernon et al., 2018):

$$I_{n}^{2}=\frac{{\left(E\left[f_{n}\left(x\right)\right]-z_{n}\right)}^{2}}{Var\left[f_{n}\left(x\right)\right]+Var\left(e_{n}\right)},$$ (1)

where $I_{n}^{2}$ is the squared implausibility measure in the input space at point **x** for n-th output feature; *f*_*n*_(**x**) is the feature value, and *E* [*f*_*n*_(**x**)] is the feature mean; *z*_*n*_ is the mean value of an experimental biomarker; *Var*[*f*_*n*_(**x**)] and *Var*(*e*_*n*_) are the simulation and observation dispersions, respectively.

For each parameter point **x**, a threshold was applied to determine whether the set of the model’s input parameters remained in the space of acceptable parameters for the current wave, as follows: if max |*I*_*n*_(**x**)| > *I*_*threshold*_, then **x** is considered implausible. We considered *I*_*threshold*_ = 3 according to Pukelsheim’s 3-sigma rule (Pukelsheim, 1994).

For *Ca* ^2+^ transient biomarkers, implausibility measures were not calculated for lack of data on biomarker mean and variability, so we used the minimum and maximum values of the biomarker as cutoff borders for the range of accepted values (Table 1).

Due to data filtering, the NROY space for the calibrated models was iteratively reduced in each wave.

#### 2.3.7 Augmentation of sampled parameter set

If less than 10^5^ points remained in the reduced parameter space as a result of model calibration, additional points were selected around each accepted point. For new point sampling, use was made of a multidimensional normal distribution centered at a given point with a covariance matrix *I* × 0.05, where *I* is the identity matrix.

In each subsequent wave, 300 new input parameter vectors for simulator calculation were sampled from the NROY space obtained in the preceding wave. Then, the algorithm is repeated from the simulator calculation step before convergence (approaching the asymptote and a reduction of the number of emulator points less than 0.1% starting from wave 47) (Supplementary Figure S1).

After 60 waves of the HM algorithm, the biomarkers of the final population of models were concentrated in the regions of experimentally observed biomarkers, displaying distributions close to the experimental ones (see Results section), which makes the modeling process very efficient. A more detailed description and justification of the method can be found elsewhere (Rasmusen and Williams, 2006; Andrianakis et al., 2015; Coveney and Clayton, 2018; Vernon et al., 2018).

#### 2.3.8 Initial population of calibrated models

Finally, we calculated 1,000 simulators with parameter vectors sampled from the restricted input space obtained in the last 60th wave. Then we calibrated this population by rejecting models with biomarkers falling outside the min-max range derived from the experimental data (Table 1) or exhibiting abnormalities in the AP or *Ca^2+^* transient waveform.

The History matching method was implemented in *Python3*, while the regression models based on Gaussian processes were implemented using *GPflow2* library. The ordinary differential equations of the simulators were solved with the help of CVODE from Sundials suite (Hindmarsh et al., 2005).

### 2.4 Mechanical tests

To evaluate further the obtained population of models, calibrated using characteristics of cellular electrophysiology and *Ca^2+^* transient, we compared simulated characteristics of the mechanical activity in the models with available experimental data (Table 1). The models which did not meet the re-calibration criteria were rejected. Then, we investigated the electro-mechanical activity in the virtual cardiomyocytes after changing the mechanical conditions of contraction.

For each model in the population, we performed two types of tests: 1) change in the initial length of the virtual cardiomyocyte (i.e., the change in the mechanical preload that stretches the cell) in the isometric twitches, when the length of the cardiomyocyte is kept constant; and 2) change in the mechanical afterload applied to the contracting cardiomyocyte in isotonic twitches under constant load. The models with abnormalities in the excitation and/or contraction under the mechanical interventions were revealed and rejected from the population.

The effect of initial length on the isometric force development was studied in experiments where three different preloads were applied to simulate experimental data from (Vahl et al., 1997; Holubarsch et al., 1998). Specifically, the initial length of the virtual muscle was step-wise reduced from 0.93*L*_max_, which is the initial length used for the TP + M model, to 0.80*L*_max_. For each model in the population, we utilized the peak force during steady-state isometric twitches (after 200 cycles at a stimulation frequency of 1 Hz) to plot the ‘length—force’ (L-F) relationship. The values of the peak isometric forces obtained at different initial lengths were normalized to the values of the isometric force obtained at a length of 0.93*L*_max_, which was different for each model of the population.

When simulating the activity of each model in the population during isotonic afterloaded contractions, we evaluated the first twitch under the imposed external load (afterload) after the baseline isometric contraction with a preload of 0.93*L*_max_. The value of the afterload was expressed in fractions of the peak isometric force (*F*_max_) developed by a given model. Three afterloads were applied: 0.25, 0.5, and 0.75 of *F*_max_. The results obtained for each model in the population for afterloaded contractions provided input data for plotting the maximum velocity of cell shortening against the afterload as a ‘force - velocity’ (F-V) relationship.

### 2.5 Drug testing

To test further our final population of accepted electro-mechanical models of the human cardiomyocyte, we simulated the effects of two drugs to assess their effect on the cell electrical and contractile activity in the models. These were Dofetilide (class III antiarrhythmic) and Verapamil (class IV antiarrhythmic) drugs with well quantified and documented action on ionic currents and thoroughly evaluated effect on AP and pro-arrhythmic risk. The two drugs are oppositely categorized with respect to the risk of Torsade de Pointes (TdP) arrhythmias. Dofetilide prolongs QT interval and is associated with high TdP risk (Jaiswal and Goldbarg, 2014; Ibrahim et al., 2021). Verapamil is an L-type *Ca* ^2+^ channel blocker and is considered safe with no TdP risk according to CredibleMeds; however, Verapamil poisoning can cause symptoms such as hypotension, AV block and bradycardia (Hofer et al., 1993; Barrow et al., 1994; Atemnkeng et al., 2021).

The effects of these drugs on ionic currents were simulated using the simple pore-block model (Brennan et al., 2009), based on *in vivo* patch-clamp measurements of IC50 values and Hill’s coefficients from (Passini et al., 2017) for Dofetilide, and from (Kramer et al., 2013) for Verapamil. Their effects on the model outputs were tested at concentrations from 0.1 to 100× EFTPCmax (maximal effective free therapeutic concentration) and compared with baseline. The experimental IC50, Hill coefficients, and drug concentrations for the pore-block model are reported in Table 2. Additionally, the effects of both drugs on a block of ion channels are shown in Supplementary Figure S2 and Supplementary Figure S3 in Supplementary Materials.

**TABLE 2:** IC50 and Hill coefficient (h), EFTPCmax values for Dofetilide (Passini et al., 2017) and Verapamil (Kramer et al., 2013) and clinical proarrhythmic risk as reported by CredibleMeds.

Compounds	IC50(h), µM				EFTPCmax, µM	TdP risk category
	INa	ICaL	IKr	IKs		
Dofetilide	31.9 (0.54)	201 (1)	0.013 (1.56)	135 (1)	0.00	1
Verapamil	32.5 (1.33)	0.2 (0.80)	0.25 (0.89)		0.09	0

## 3 Results

### 3.1 Initial population of electro-mechanical models

**FIGURE 1:**
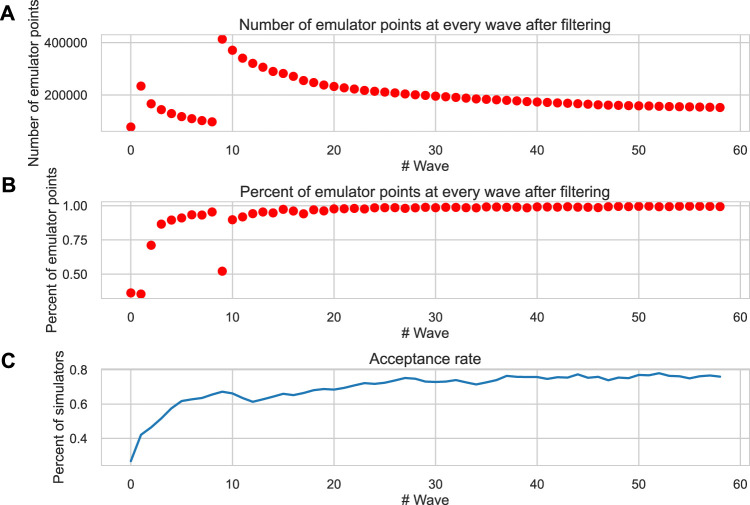
Convergence of the HM algorithm. **(A)** The number of permissive parameter sets from the current parametric space after calibration of emulators at each wave of the HM algorithm. **(B)** The ratio of permissive emulators in the current wave with respect to the previous one. **(C)** The acceptance rate is the proportion of calculated models (simulators) that meet the calibration criteria (Table 1) in each wave. The *X*-axis is the wave number. In the seventh wave, additional points are generated (see the description of the HM procedure in the “Methods” section).

A population of electro-mechanical models of human cardiomyocytes was created based on the reference TP + M model (Bazhutina et al., 2021) assuming randomly varying 11 input parameters over a wide range, using a scaling factor of the reference value ranged from 0 to 2 for each parameter. The initial electrically calibrated population of cardiomyocyte models was generated using the History matching (HM) method. Figure 1A shows the number of sampled points (parameter vectors or parameter sets) in the space of varying model parameters in each iterative wave after calculating the emulators and filtering them by AP and *Ca* ^2+^ transient biomarkers (Table 1, Calibration step). Since less than 10^5^ points remained in the permissive parameter space as a result of emulator output calibration after the 7th wave, additional points were sampled. Over the subsequent iterations, the number of points remaining in the permissive parametric space for calibrated emulators during each HM wave approached an asymptotic limit of ∼ 150,000 sets after a 50-wave run.

The percentage of points rejected via the calibration of emulator outputs rapidly decreased down to < 1% of the tested emulators, so that about 100% of emulators met the calibration criteria at the last iterations (Figure 1B). However, not every parameter set predicting acceptable outputs from the emulator (regression model) also produced acceptable outputs from the simulator (ODE model) calculated for a given parameter set. The acceptance rate, that is, the fraction of simulator models meeting the calibration criteria (Table 1), increased with each HM iteration from 20% up to about 80% at the last 60th HM wave (Figure 1C). This acceptance rate outperforms the indicators obtained by alternative methods (for example, an acceptance rate of 40% in (Passini et al., 2017)).

After the last 60th wave of the HM algorithm, we again sampled 1,000 points from the reduced parametric space predicted as permissive from the emulator model, calculated simulator models for each sampled parameter set, and once again performed the calibration of the simulator outputs. The models generating biomarker values for AP and *Ca* ^2+^ transients outside the experimental data range (Table 1) and/or demonstrating repolarization abnormalities were automatically rejected from the population of models. Examples of such rejected models are shown in Supplementary Figure S4. The excitation abnormalities we observed in the rejected models included various types of disturbances, such as early afterdepolarizations (EADs), delayed afterdepolarizations (DADs), premature APs and extrasystoles and failed contractions.

Finally, an initial population of electrically calibrated models consisting of 769 models was obtained.

### 3.2 Re-calibration of the population of models using mechanical tests

Since the initial population of models was calibrated using only AP and *Ca* ^2+^ transient characteristics and no “mechanical” biomarkers of the force and contraction generated by the electro-mechanical models were evaluated during parameter sampling in the HM algorithm, the population was then further re-calibrated.

**FIGURE 2:**
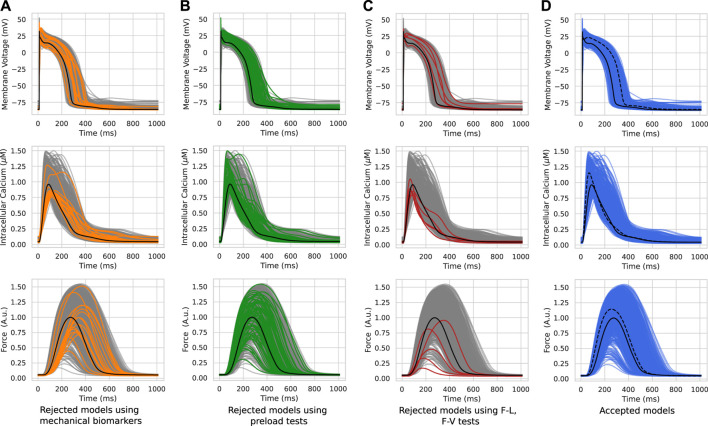
Model re-calibration using mechanical tests. Action potentials (upper panels), *Ca* ^2+^ transients (middle panels) and active force generated by models (bottom panels) from the initial population re-calibrated step-by-step using mechanical tests: the calibration using the force biomarkers **(A)**; the calibration during preload tests when models were rejected due to excitation abnormalities that occurred at reduced initial cell lengths **(B)**; the calibration when models were rejected due to abnormalities in the ‘length - force’ and ‘force - velocity’ dependencies **(C)**. The colored lines in panels A–C indicate the rejected models in the respective calibration step. The gray lines indicate models meeting calibration criteria at a current re-calibration step. The blue lines show the final population of accepted models **(D)**. The black line indicates the reference TP + M model. The dashed line on the right panel represents one of the accepted models. Force is normalized to the peak isometric force (*FT*_*peak*_) of the reference TP + M model.

Figure 2 shows several steps in the re-calibration of the initial population of models using mechanical biomarkers and tests with different mechanical conditions during cell contractile cycles (Table 1, Re-calibration stage). The colored lines in the figure show AP, *Ca* ^2+^ transient and force waveforms in the models rejected at each mechanical calibration step. Gray lines show the output signals from acceptable models which passed the mechanical tests during re-calibration.

#### 3.2.1 Mechanical biomarkers

At the first step of mechanical calibration of the models, 17 models that did not meet the calibration criteria for the force based on the experimental cellular mechanics data (Table 1, Re-calibration stage) were rejected from the initial population of 769 models (Figure 2A). Although the waveforms of the AP, *Ca* ^2+^ transients and force fell within the entire cloud of simulated signals in the population, the rejected models had *FTTP* values (the time to reach the peak isometric force) that were too large.

#### 3.2.2 Preload tests

In the calculation of models for HM waves and the calibration of the initial population, we utilized a reference initial length of 0.93*L*_max_ with a corresponding initial sarcomere length of 2.1 *μ*m. In the next step of mechanical calibration, each of the 752 models with acceptable mechanical biomarkers at the reference initial length, was evaluated at three reduced preloads (initial lengths) for cell isometric twitches, which provided shorter initial cell lengths of 0.90, 0.85, and 0.80*L*_max_ in descending order.

**FIGURE 3:**
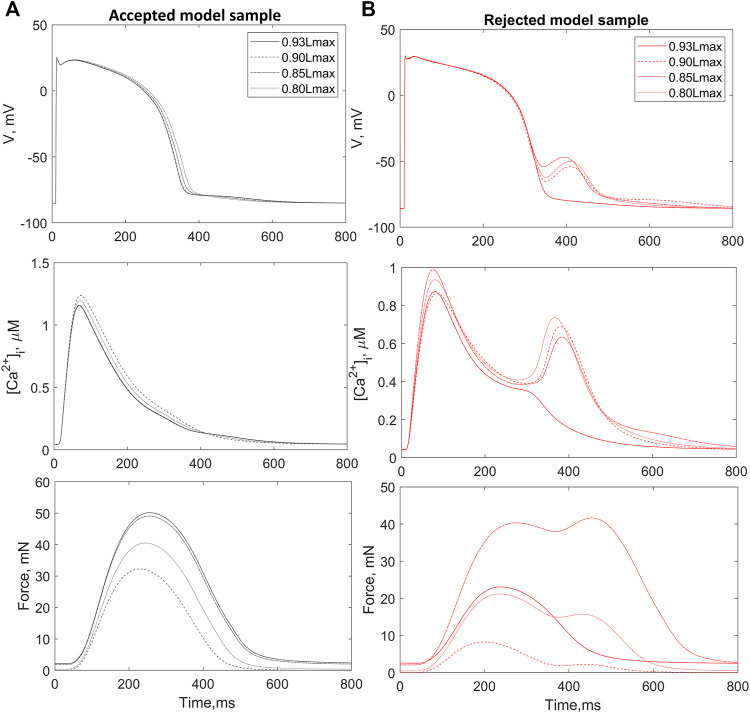
Preload tests. Examples of an accepted **(A)** and rejected **(B)** model under re-calibration of the models using variation in the initial length during isometric contractions. The rejected model produces excitation abnormalities at reduced preloads. The figure shows action potential (AP, upper panels), *Ca* ^2+^ transients ( ${\left[Ca^{2+}\right]}_{i}$ , middle panels) and active force (Force, bottom panels) generated by the virtual cardiomyocyte during steady-state isometric twitches at different initial lengths.

A total of 73 out of the 752 population models were rejected from the population due to depolarization and/or repolarization abnormalities for at least one of the tested initial lengths (Figure 2B). Examples of accepted and rejected models at this mechanical calibration step are shown in Figure 3. In consistency with the experimental data, the accepted models demonstrate an essential reduction in the peak force and duration of the contractile cycle at reduced afterloads and corresponding initial lengths. Decreased mechanical preloads and related changes in the mechanical activity of contracting cells result in an increase in the AP and *Ca* ^2+^ transient in the models. It should be noted that, like to the accepted models, the models rejected at the preload testing step showed normal behaviour at the reference preload and initial length (Figures 2B, 3). However, the reduction in preload caused repolarization abnormalities in the rejected models (Figure 3), induced by spontaneous *Ca* ^2+^ release from the SR, resulting in abnormal double-peak contractions.

#### 3.2.3 L-F curves

**FIGURE 4:**
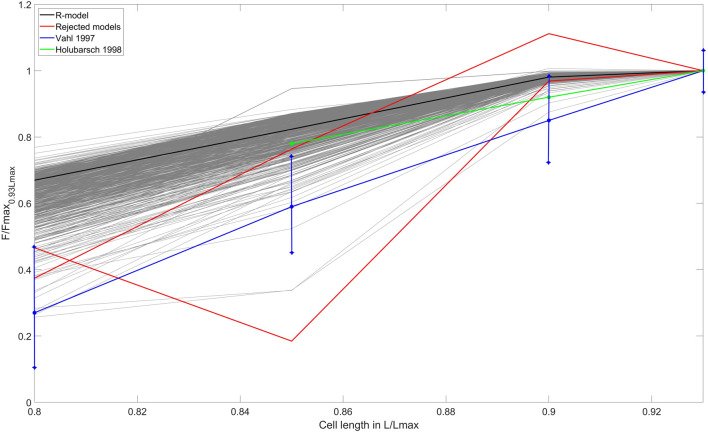
‘Length – force’ (L-F) relationship test. The L-F curves were obtained during re-calibration by preload tests of the population that consisted of models that had no excitation abnormalities. The L-F diagram shows the dependence between the initial cell length and the isometric peak force developed. The curves derived from experimental data on human cardiac preparations are shown by the blue line (Vahl et al., 1997) and the green line (Holubarsch et al., 1998). The L-F dependence for the reference TP + M model is shown by the solid black line. The models rejected after the preload tests are shown in red lines. The *X*-axis indicates the initial length of the virtual cell, normalized to *L*_max_ (the length at which the cell develops maximum isometric force). The *Y*-axis represents the isometric peak force at the corresponding length, normalized to the value of the isometric peak force at an initial length of 0.93*L*_max_, which is different for each model of the population.

Next, we evaluated the ‘length - force’ (L-F) dependence between the initial cell length and the peak isometric force at this length in the models that passed the preload tests (Figure 4). Experimental data on the L-F relationship in human are limited (Table 1), and we could only find two studies with such data (Vahl et al., 1997; Holubarsch et al., 1998) (shown in Figure 4). Two more works reported data on the L-F relationship in human, though only when cardiomyocytes are stretched (Brandenburger et al., 2012; Milani-Nejad et al., 2015). To validate models using the isometric L-F relationship, the following qualitative feature should be fulfilled: the peak force must increase with length. Among the 679 models, we found only 2 that did not demonstrate a monotonous increase in force with increasing length and rejected them from the population (Figure 4, red lines). An example of the output signals in one of the rejected models as compared to an accepted model is shown in Supplementary Materials (Supplementary Figure S5).

#### 3.2.4 Afterload tests

**FIGURE 5:**
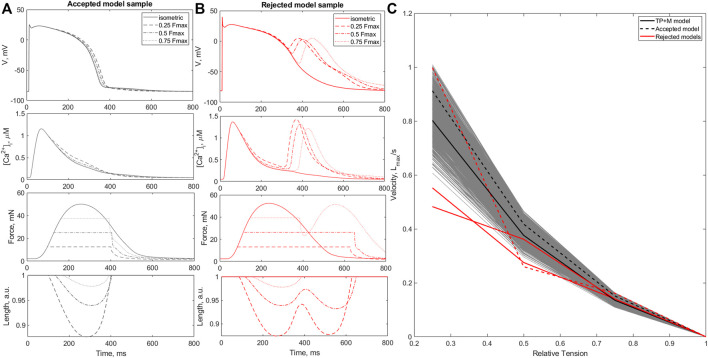
Afterload tests. Panels **(A, B)** show examples of an accepted **(A)** and a rejected **(B)** model during afterload tests. The panels show action potentials (AP), *Ca* ^2+^ transients $\left({\left[Ca^{2+}\right]}_{i}\right)$ , force and length (normalized to initial length) during isotonic contractions at different afterloads (0.25, 0.5 and 0.75 of *F*_max_, where *F*_max_ is the isometric peak active force). **(C)** ‘Force - velocity’ (F–V) relationships in the models calibrated during the afterload tests. The black line shows the F-V relationship for the reference TP + M model. The F-V curve for the accepted model in panel A is indicated by the black dashed line. The F-V curves for all models rejected in the afterload tests are shown by red lines, with the dashed line corresponding to the model shown in panel **(B)**. The *X*-axis indicates the afterload, normalized to the respective *F*_max_. The *Y*-axis indicates the velocity of shortening of cardiomyocytes during afterloaded isotonic twitches.

The next step in the mechanical calibration of the models that passed all previous mechanical tests was performed with isotonic contractions at various afterloads from the physiological range of 0.25–0.75 of the peak isometric force *F*_max_ recorded during heavy-loaded contractions. Three models out of 677 models were found to exhibit repolarization abnormalities during isotonic contractions at low afterloads (Figure 5B). The ‘force-velocity’ (F-V) dependencies of these models also showed atypical trends compared to the overall population (Figure 5C). The models were also rejected from the calibrated population.

While the accepted models showed the characteristic features of afterloaded contractions observed in experiments (Sonnenblick, 1962), i.e., an increase in amplitude and velocity of shortening with a decrease in applied afterload (Figure 5A), in the rejected models afterload reduction led to spontaneous *Ca* ^2+^ release from the SR followed by EADs and double-peaked prolonged contractions (Figure 5B).

**FIGURE 6:**
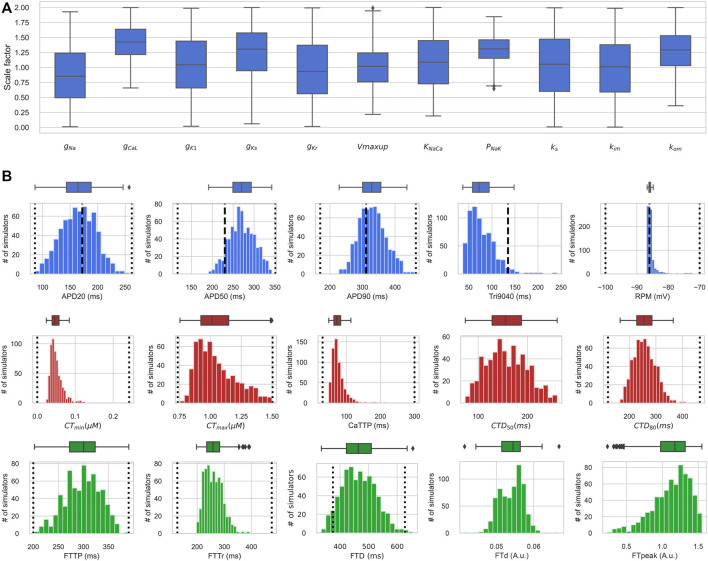
Distribution of input parameters and output biomarkers in the electro-mechanically calibrated models. **(A)** Scaling factors of input parameters in accepted models from the final population, shown as boxplot diagrams. The middle marker of the boxes shows the median, the box boundaries are the 25th and 75th percentiles, and the whiskers extend to the most extreme data points. **(B)** Boxplots (upper) and histograms (lower) of the distribution of biomarker values of action potential, *Ca* ^2+^ transient and isometric force in the population of accepted electro-mechanical models. The dotted vertical lines indicate the minimum and maximum values of the biomarkers derived from experimental data (Table 1), and the dashed vertical lines indicate the experimental mean values of the AP biomarkers (Table 1). The *Y*-axis indicates the number of models, and the *X*-axis the biomarker value. *RMP* is the resting membrane potential; *APD*_*xx*_ is the duration of AP at the XX% level of repolarization; *Tri*_9040_ - triangularity of AP, defined as the difference between *APD*_90_ and *APD*_40_; *CT*_min_, *CT*_max_ are diastolic and systolic values of the concentration of free *Ca* ^2+^ in the cytosol; *CaTTP* is the time to reach the peak of the *Ca* ^2+^ transient; *CTD*_*xx*_ is the duration of the *Ca* ^2+^ transient at the XX% decay level; *FTTP* is the time to reach isometric peak force; *FTTr* is the time of 90% of isometric force decay; *FTD* is the isometric twitch duration; *FT*_*d*_ and *FT*_*peak*_ are diastolic and maximum systolic force levels. Both *FT*_*d*_ and *FT*_*peak*_ are normalized to the *FT*_*peak*_ of the reference TP + M model.

After all mechanical tests, a final population of 674 electro-mechanical models of the human cardiomyocyte out of total 769 in the initial population was calibrated and validated in a series of electrophysiological and mechanical tests. The models in the final population of accepted models produced of AP, *Ca* ^2+^ transient and force/shortening output signals of normal waveform shape and duration. The physiologically relevant biomarkers (amplitude, time to peak, time to a certain percentage of relaxation, *etc.*) (Figure 6) occured in the range of values representing the natural variability of the characteristics in agreement with reported experimental data in human ventricular cardiomyocytes. Importantly, in each model of the population, the assorted output characteristics are consistent with each other, and their combination is consistent with the experimental data. Moreover, each model reproduces a qualitatively adequate response to a change in the mechanical conditions of cell contraction, such as a change in the initial sarcomere length and a change in the afterload imposed on the cell. Note that the reference TP + M model also passed all of our electro-mechanical tests and was included in the final population of accepted models.

### 3.3 Input parameters in accepted and rejected models

#### 3.3.1 Distribution of input parameters in the population of accepted models

Figure 6A shows distributions of scaling factors for variable input parameters in the final population of electro-mechanically calibrated models. It is seen that 6 out of the 11 parameters are distributed around the reference values (mean scaling factor is near 1), while the remaining 5 parameters have average scaling factors that differ significantly from 1 (Table 3).

**TABLE 3:** Summary of statistics on the distribution of scaling factors for model parameters in the population of electro-mechanically calibrated models.

	*g*_*Na*_	*g*_*CaL*_	*g*_*K*1_	*g*_*Ks*_	*g*_*Kr*_	*V*_*maxup*_	*K*_*NaCa*_	*P*_*NaK*_	*k*_*s*_	*k*_*im*_	*k*_*om*_
mean	0.87	1.42	1.05	1.25	0.97	1.01	1.10	1.3	1.03	0.99	1.28
std	0.46	0.29	0.48	0.43	0.49	0.34	0.45	0.23	0.52	0.49	0.35
min	0.01	0.66	0.02	0.06	0.02	0.22	0.19	0.64	0.01	0.01	0.36
25%	0.50	1.22	0.66	0.95	0.56	0.76	0.73	1.15	0.6	0.59	1.03
50%	0.85	1.43	1.05	1.31	0.93	1.02	1.09	1.31	1.06	1.01	1.29
75%	1.24	1.64	1.44	1.58	1.37	1.24	1.45	1.46	1.47	1.38	1.53
max	1.93	2.00	1.99	2.00	1.99	1.99	2.00	1.85	1.99	1.99	2.00

The varying parameters in the accepted models have normal distributions with high variability (*σ* of about 0.5 for the parameter scaling factor) within the diapason of population sampling. Overall, the 25% percentile for the parameter scaling factor is higher than 0.5, and the 75% percentile is lower than 1.6 for every varying parameter in the population (Figure 6; Table 3). Surprisingly, the maximum permissive value of the scaling factor is close to the upper bound of 2.0 for each parameter tested. At the same time, the minimum permissive value is not near zero for several parameters. Particularly, the minimum values are higher than 0.6 for *g*_*CaL*_ and *P*_*NaK*_, near 0.3 for *k*_*om*_, and near 0.2 for the maximum velocity *V*_max_ of SERCA pump and *K*_*NaCa*_ of the NCX current, suggesting more stringent restrictions for these essential ionic parameters affecting markedly the characteristics of AP and *Ca* ^2+^ transient in normal cells.

#### 3.3.2 Correlation between input parameters in accepted models

We analysed the correlations between the input parameters in the accepted models to understand whether they are related to each other and whether they need to be sampled according to the dependencies revealed in a population of models for normal cardiomyocytes. Supplementary Figure S6 shows a scatter diagram of the varying input parameters in pairs (lower triangular part). The corresponding values of Pearson’s correlation coefficient and *p*-values between the parameters in the population of accepted models are given in the upper triangular part.

**FIGURE 7:**
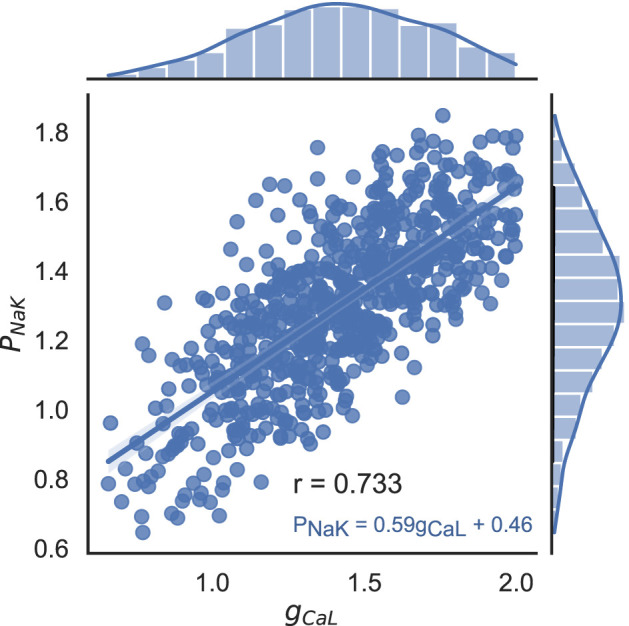
Correlation between scaling factors for parameters *P*_*NaK*_ and *g*_*CaL*_ in the final population of accepted models. Dots indicate values in individual models, histograms show the marginal distribution of the corresponding parameters, and the blue line shows a linear regression line with the equation *P*_*NaK*_ = 0.59 ⋅ *g*_*CaL*_ + 0.46. Pearson’s correlation coefficient r = 0.733, *p* < 0.001.

Most of the model input parameters do not correlate with each other, except for the pair of maximum NKX current (*P*_*NaK*_) and maximum conductivity of the L-type *Ca* ^2+^ current (*g*_*CaL*_), which display a strong positive correlation (r = 0.73, p < 0.001) (Figure 7). In the physiological range, these currents must be balanced to provide physiologically acceptable intracellular *Ca* ^2+^ and *Na* ^+^ concentrations. The current via NKX is largely determined by the gradient of *Na* ^+^ concentration, and it itself defines the *Na* ^+^ level inside the cell. A deficiency in NKX current may cause an elevation in the intracellular *Na* ^+^ level, which in turn activates the NCX. The latter is characterized by the level of intracellular *Ca* ^2+^ concentration, which largely depends on the *Ca* ^2+^ current through L-type *Ca* ^2+^ channels (*I*_*CaL*_). Thus, a small *P*_*NaK*_ can lead to an increase of *Na* ^+^ inside the cell, and an enhanced *Ca* ^2+^ current via the reverse mode of NCX together with a high *I*_*CaL*_ (with a large *g*_*CaL*_) cannot be balanced by the forward NCX, which leads to an increase in the *Ca* ^2+^ level in the cell. As a result, we get either too high and prolonged a *Ca* ^2+^ transient the characteristics of which do not fall within the acceptable range of biomarkers, or *Ca* ^2+^ overload leading to spontaneous *Ca* ^2+^ releases from the SR and abnormalities in repolarization. Thereafter, models with imbalanced high *g*_*CaL*_ and low *P*_*NaK*_, or *vice versa*, do not fall into the physiologically acceptable population.

#### 3.3.3 Input parameters: accepted vs. rejected models

We compared the subsets of varying input parameters selected from the non-implausible parameter space in the cohorts of models that were finally accepted and rejected, either according to the calibration criteria for the AP and *Ca* ^2+^ transient in the last step of the HM algorithm or during recalibration using the mechanical tests (see parameter distributions in the groups shown in Supplementary Figure S7A). Here, we used the Wasserstein distance (WD) to assess the difference between the input parameter distributions in the accepted and rejected models for each varying parameter (Supplementary Figure S7B). The largest WD was found for the distributions of the maximum rate of *Ca* ^2+^ uptake into the SR (*V*_*maxup*_), which was several times higher than the WD for other parameters, suggesting the contribution of this parameter value to the inconsistency of simulated biomarkers with calibration criteria and/or occurrence of disturbances in the electro-mechanical activity observed in the rejected models.

In Supplementary Figure S6 we demonstrate the superposition of pairwise scatter diagrams for the input parameters in the groups of finally accepted models (blue colour) and the models rejected during the mechanical tests (red colour). The scatter plots also reveal intersection of the parameter areas in the accepted and rejected models, and the uni-parametric (shown on the diagonal in Supplementary Figure S6) almost overlap with each other in the accepted and rejected models, not allowing for the mechanistic distinction between the groups. The range for each parameter in the final population is contiguous. However, this does not mean that any random combination of parameter values from the parameter space built on these contiguous intervals will yield an accepted model. Some combinations of parameter values from acceptable intervals may yield models that do not meet the criteria for electrophysiological biomarkers or mechanical tests, particularly because some parameters are physiologically closely related (and correlated) and their values cannot be set arbitrarily. In Section 4.2, we have discussed why the parameters for the rejected models (shown on the scatterplot in Supplementary Figure S6) lie within the projection area of the accepted points.

**FIGURE 8:**
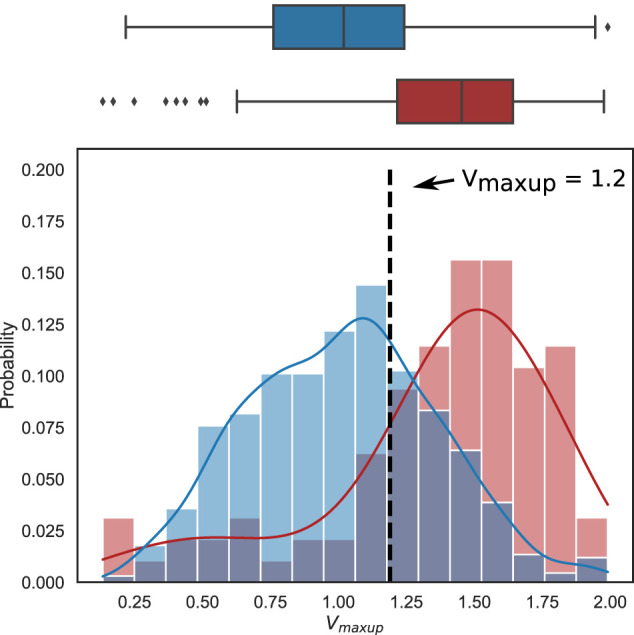
Difference in the distributions of the scaling factor for the maximum rate of the SR SERCA pump (*V*_*maxup*_) between the accepted models (blue) and the models rejected from the electrophysiologically calibrated population using mechanical testing (red). Boxplots (top) and histograms (bottom) show the *V*_*maxup*_ scaling factor distribution in the population of accepted (blue) and rejected (red) models. Each histogram was normalized so that the column area be equal to 1. The vertical dotted line marks the threshold for *V*_*maxup*_, above which anomalies in the cell models are predicted in mechanical tests (see Results for details).

Analysis of the marginal distributions for each input parameter in the accepted and rejected models also showed no significant difference between the mean parameter values in the accepted and rejected models with the only exception of *V*_*maxup*_ (Supplementary Figure S7A). The histograms of *V*_*maxup*_ distribution in the accepted models and models rejected during mechanical tests are shown in (Figure 8). The mean *V*_*maxup*_ in the accepted models is close to the *V*_*maxup*_ in the reference TP + M model (scaling factor is near 1: 1.01 ± 0.34), while in the rejected models mean scaling factor for *V*_*maxup*_ is about 1.4 times larger (1.36 ± 0.42). Moreover, we found an increasing ratio of the rejected models with increasing the *V*_*maxup*_ scaling factor over the reference value (Supplementary Figure S8). Note, in the models rejected via electrophysiology calibration criteria after the last HM wave, mean scaling factor for *V*_*maxup*_ is higher than that for mechanically rejected models (1.46 ± 0.39), and the WD between the distributions of *V*_*maxup*_ in accepted and rejected models is also higher for the models rejected via electrophysiology tests (0.46 vs. 0.36, respectively, see Supplementary Figure S7).

As we were not able to find deterministic conditions to distinguish model parameters between accepted and rejected models, we applied machine learning to predict the likelihood of model rejection in the non-implausible parameter space we found using the HM approach. First, we used a logistic regression to range contribution of input parameters to the score of model rejection. Supplementary Figure S9 shows the relative importance of the input parameters for models classification into rejected (class 1) or accepted (class 0) classes. Not surprisingly, we found that *V*_*maxup*_ has the greatest contribution to the classification score among other parameters increasing the score with the higher parameter value.

Following the above analysis we developed uni-parametric logistic regression classifiers to classify the accepted and rejected models using the *V*_*maxup*_ as the only input parameter. A logistic regression classifier was able to predict a model rejection via electrophysiology tests with a high accuracy of 0.73 (ROC AUC of 0.79, sensitivity of 0.79 and specificity of 0.71). Similar performance was demonstrated by a classifier predicting a model rejection under the mechanical tests with an accuracy of 0.71 (ROC AUC of 0.77, sensitivity of 0.77 and specificity of 0.70). In the latter case, the analysis revealed a threshold of *V*_*maxup*_ with a scaling factor of 1.2 that statistically separated the accepted and rejected models. The odds ratio of having an accepted model when the scaling factor was less than 1.2 versus when the scaling factor was greater than 1.2 was 8.1 (95% confidence interval [4.9, 13.5]), showing the high power of this prediction. The probability of finding a normal model in the case of a scaling factor of *V*_*maxup*_ lower than 1.2 was 95% high. In the case of a scaling factor higher than the threshold, the probability to sample a normal model is reduced to 70% but still suggesting a high acceptance rate for models with parameters from the non-implausible parameter space we found.

It should be noted, that in contrast to the models rejected during the initial AP and *Ca* ^2+^ transient calibration, the models rejected during the mechanical tests have realistic AP, *Ca* ^2+^ transient and force waveforms under the baseline mechanical conditions similar to the accepted models (Figure 2). This shows the importance of the mechanical tests and variation in the contraction conditions when using electro-mechanical model populations.

### 3.4 Distribution of output biomarkers in the population of accepted models

Figure 6B demonstrates the distribution of output biomarkers derived from simulated AP, *Ca* ^2+^ transient, and isometric force signals in the final population of accepted models. According to the calibration criteria, all output biomarkers in the accepted models fall within the acceptable range consistent with the experimental data (Figure 6B; Table 4, compare with the experimental data from Table 1). In the HM procedure, only AP biomarkers were calibrated according to their parameters of variability in the experimental data (mean, standard deviation), so the average values of *APD*_20_, *APD*_90_, and *RMP* are close to the average values obtained in the experiments (Figure 6B). The mean of *APD*_50_ is larger and *Tri*_9040_ is smaller in the model population than in the experimental data, while remaining between minimum and maximum values.

**TABLE 4:** Summary of statistics for the distribution of output biomarkers of AP, calcium transient and force generated by the accepted models in the final population.

	*APD*20(*ms*)	*APD*50(*ms*)	*APD*90(*ms*)	*Tri*9040(*ms*)	*RMP*(*mV*)
mean std	164.84	270.68	330.21	78.84	−85.56
	30.93	30.14	40.93	29.02	1.48
	*CT*_min_(*μM*)	*CT*_max_(*μM*)	*CaTTP*(*ms*)	*CTD*_50_(*ms*)	*CTD*_80_(*ms*)
mean std	0.05	1.04	75.35	155.34	257.2
	0.016	0.15	18.86	41.11	39.63
	*FTTP*(*ms*)	*FTTr*(*ms*)	*FTD*(*ms*)	*FTd*(*A*.*u*.)	*FT*_*peak*_(*A*.*u*.)
mean std	298.15	261.08	466.09	2.36	49.35
	34.95	33	58.12	0.14	11.26

Model calibration using *Ca* ^2+^ transient biomarkers was limited by minimum and maximum values for lack of a sufficient pool of experimental data to obtain a representative distribution of *Ca* ^2+^ transient characteristics in human cardiomyocytes. However, *Ca* ^2+^ transient in our accepted models yielded mean values for diastolic and systolic concentrations of 0.05 ± 0.016 *μM* and 1.05 ± 0.15 *μM* (see *CT*_min_, *CT*_max_ in Table 4), which are consistent with experimental data in human ventricular preparations. The mean values for the temporal characteristics of *Ca* ^2+^ transients in the accepted models also agree with the experimental data (see time to peak *CaTTP* and duration *CTD*_*XX*_ at the XX% decay level). Note that *CT*_min_, *CT*_max_ and *CaTTP* have asymmetric distributions with the most frequent values closer to the lower border of the distribution.

Figure 6B also shows the distribution isometric force biomarkers (*FTTP* is the time to reach peak isometric force, *FTTr* is time to force decay to 90%, *FTD* is the duration of the isometric twitch, *FT*_*d*_ and *FT*_*peak*_ are diastolic and peak systolic force values), which were not calibrated in the HM procedure. They were first used after the convergence of the HM algorithm for re-calibrating the initial population of models calibrated using electrophysiology and *Ca* ^2+^ transient data. It should be noted that only a few models (17 out of 769) from the initial population were rejected at this stage of mechanical calibration. This indicates that the majority of models with acceptable AP and *Ca* ^2+^ transient characteristics produce contractions that are in agreement with the experimental data, thus demonstrating the validity of our approach. Every force characteristic in the final accepted population is also within the range of minimum and maximum values available from experimental data (Figure 6; Table 4). However, data on the mechanical characteristics are very limited while those available were registered in experimental observations on a small number of preparations (n = 11 (Brixius et al., 2001), n = 8 (Vahl et al., 1998); n = 9 (Pieske et al., 1996)). Thus, the uncertainty in the force calibration data is higher compared to the data for the AP and *Ca* ^2+^ transient. Therefore, we also evaluated model contraction characteristics based on data from experimental animals. For example, our models gave a ratio of active to passive force consistent with that observed experimentally in human and is specific to normal myocardium (Vahl et al., 1997; Rossman et al., 2004; Chung et al., 2019).

#### 3.4.1 Correlations between output biomarkers in the accepted models

Supplementary Figure S10 shows pairwise scatter diagrams of the output biomarker values in the population of accepted models. In addition to the rather obvious correlations of temporal AP biomarkers with each other, there is a high correlation of force biomarkers with temporal *Ca* ^2+^ biomarkers. For example, isometric force duration (*FTD*) correlates with *Ca* ^2+^ transient duration (*CTD*_50_ and *CTD*_80_) with r = 0.75 (*p* < 0.01) and r = 0.65 (*p* < 0.01), respectively.

Moreover, the peak isometric force biomarker *FT*_*Peak*_ correlates with most of the amplitude and temporal biomarkers of *Ca* ^2+^ and force, thus yielding, for instance, r = 0.79 with *Ca* ^2+^ transient amplitude (*CT*_max_) and r = 0.62 with *CTD*_50_.

These results show tight coordination between characteristics of *Ca* ^2+^ transient and force generation in accepted models, producing simultaneously acceptable characteristics of the processes of excitation-contraction coupling, which confirms again the validity of the models.

### 3.5 Validation of the population of models

#### 3.5.1 Validity of the reference electro-mechanical TP + M model

Our population of human cardiomyocyte models was created based on the reference TP + M model (Bazhutina et al., 2021). The final population consisted of accepted models that were step-by-step calibrated using the electro-mechanical tests as discussed above. The reference model met all these calibration criteria. Reference value (scaling factor of 1) for each of 11 input parameters varying in the population falls into the permissive parameter space and a majority of the referent parameters lies between the 25% and 75% percentiles of the parameter distribution in the final population. Only for two parameters, the conductivity of the inward L-type *Ca* ^2+^ current *g*_*CaL*_ and the maximum density *P*_*NaK*_ of the NKX current, the reference values are lower than the 25% percentile (but higher than the mean-2*σ* margin) for the population distribution, with their means being higher (by 40% for *g*_*CaL*_ and by 30% for *P*_*NaK*_) than the reference values. These observations confirm that our reference model is representative of the population of calibrated models and can be used in various applications which do not require using the population approach.

#### 3.5.2 Response to modulations of ionic currents

Models from the final population of accepted models, including the reference TP + M model, were evaluated using additional experimental data on human myocardium response to various interventions which were not used for model calibration. We evaluated the sensitivity of the models to variations in several ionic currents, the qualitative effects of which on AP characteristics have been experimentally assessed in human. The conductances of the following nine ionic currents were modulated using a scaling factor of 0.5 (two times reduction) or 2.0 (two times evaluation) as compared to baseline: fast *Na* ^+^ current (*I*_*Na*_), L-type *Ca* ^2+^ current (*I*_*CaL*_), transient outward *K* ^+^ current (*I*_*to*_), rapid and slow delayed outward rectifier *K* ^+^ currents (*I*_*Kr*_, *I*_*Ks*_), inward rectifier *K* ^+^ current (*I*_*K*1_), *Na* ^+^ − *Ca* ^2+^ exchanger (*I*_*NaCa*_), *Na* ^+^ − *K* ^+^ pump (*I*_*NaK*_), and SERCA pump (*I*_*up*_). We assessed the uniparameteric sensitivity of the following AP biomarkers: rest membrane potential (*RMP*), peak voltage (*V*_*peak*_), maximum upstroke velocity (*dV*/*dt*_max_) and APD at 90% repolarization (*APD*_90_), in the same way as in (Riebel et al., 2021). Relative sensitivity to parameter variation was calculated as described in (Romero et al., 2009).

Supplementary Figure S11 shows the relative sensitivities of the AP biomarkers to variations in the parameters of the ionic currents in the reference TP + M model and in a representative model from the final accepted population with mean values of the parameters in the population (hereinafter referred to as a “mid-range” model). A twofold decrease in *g*_*Na*_ causes a considerable decrease in *dV*/*dt*_max_ (−27% and −17% in the TP + M and mid-range models) and *V*_*peak*_ (−20% and −4%, respectively). The model response is qualitatively consistent with the observed negative effects of sodium blockers on the membrane depolarization (Bhattacharyya and Vassalle, 1982; Legrand et al., 1983; Gottlieb et al., 1990). The *V*_*peak*_ decreases also with decreasing *g*_*NaK*_ and *g*_*CaL*_ and with increasing *g*_*to*_ or *K*_*NaCa*_.

The AP biomarkers were also essentially sensitive to variation in *g*_*Na*_, *g*_*CaL*_, *g*_*Kr*_, *g*_*Ks*_, *g*_*K*1_ and *P*_*NaK*_ (Supplementary Figure S11A, B). Specifically, an essential *APD*_90_ shortening was observed with increasing the *K* ^+^ currents: -18%, −11%, −13% in the TP + M model, and −20%, −11%, −9% in the mid-range model for a two-fold increase in *g*_*Kr*_, *g*_*Ks*_ and *g*_*K*1_, respectively. In consistency with experimental data in human cardiomyocytes, the simulations show that a decrease in *g*_*CaL*_ results in a *APD*_90_ shortening (Dangman et al., 1982; Li et al., 1999), whereas a decrease in *g*_*Kr*_, *g*_*Ks*_ and *g*_*K*1_ results in *APD*_90_ prolongation (Bosch et al., 1998; Hondeghem et al., 2001; Jost et al., 2005; Guo et al., 2008).

Thus, we showed that our calibrated models are able to qualitatively predict changes in AP as a result of modulating the activity of ionic currents.

#### 3.5.3 Drug-induced effects

To validate our final population of electro-mechanically calibrated models of human cardiomyocytes, we simulated the effects of two drugs: Dofetilide and Verapamil.

**FIGURE 9:**
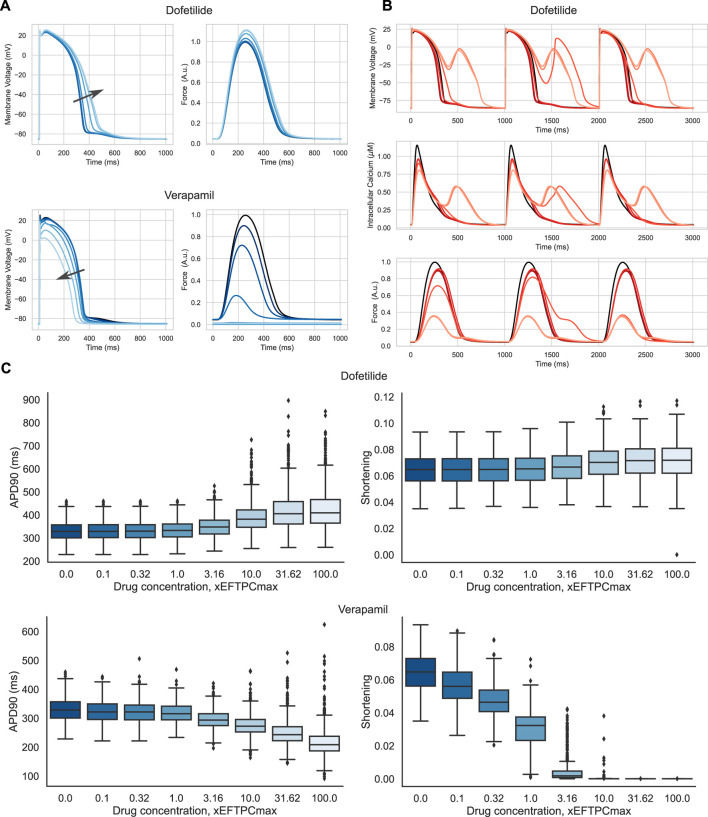
Drug effects in the population of accepted models. **(A)** Action potential and isometric force of a model sample from the final population under different concentrations of Verapamil and Dofetilide. The model that has no anomalies under drug exposure has been selected. The concentrations are coded in shades of blue, from dark blue, corresponding to baseline (zero concentration), to light blue, corresponding to 100 times the maximum effective free therapeutic concentration (EFTPCmax). The arrows indicate the direction of the changes in action potential duration with increasing drug concentration. Force is normalized to the peak isometric force (*FT*_*peak*_) of the reference TP + M model. **(B)** Action potential, *Ca* ^2+^ transient and isometric force of a model sample under different concentrations of Dofetilide. The model showing repolarization abnormalities was selected. Concentrations are coded in shades of red, from dark red, corresponding to baseline (zero concentration), to light red, corresponding to 100xEFTPCmax. At high concentrations, the model shows repolarization abnormalities in the form of additional peaks in the repolarization phase of AP and additional *Ca* ^2+^ peaks. **(C)** Boxplot diagrams showing the effects of Dofetilide and Verapamil on action potential duration (APD90) and isotonic cell shortening in the final population as a function of concentration. The solid horizontal line in each box indicates the median, and the boxes indicate the first and third quartiles for the parameter values. The whiskers indicate either 1.5 times of the interquartile range or the furthest data points.

Figure 9A shows examples of the drug action on the AP waveform and isometric force in a representative mid-range model from the final accepted population. The drugs produced an opposite effect on AP duration. AP prolongs in simulations of Dofetilide action on the ionic currents, while AP reduces under Verapamil. At concentrations higher than EFTPCmax, Dofetilide causes repolarization abnormalities in a number of models from our population. Figure 9B illustrates an example of EADs occurring under Dofetilide in such models from the final population. These results are qualitatively consistent with experimental data on human ventricular trabeculae (Page et al., 2016), ventricular myocytes (Guo et al., 2011), stem cell-derived cardiomyocytes (Gibson et al., 2014; Lu et al., 2015; Zeng et al., 2016). At the same time, Dofetilide does not affect the isometric force of the virtual cell, in line with no data reported on the effect of Dofetilide on myocardium contractility. On the contrary, as its concentration is increased, Verapamil causes a crucial reduction in force production, which also agrees with experimental data for humans (Nguyen et al., 2017). Figure 9C summarises the effects of increasing the concentration of the drugs on AP duration and amplitude of cell shortening in isotonic contractions under an afterload equal to half the maximum isometric force in the population of accepted models.

**FIGURE 10:**
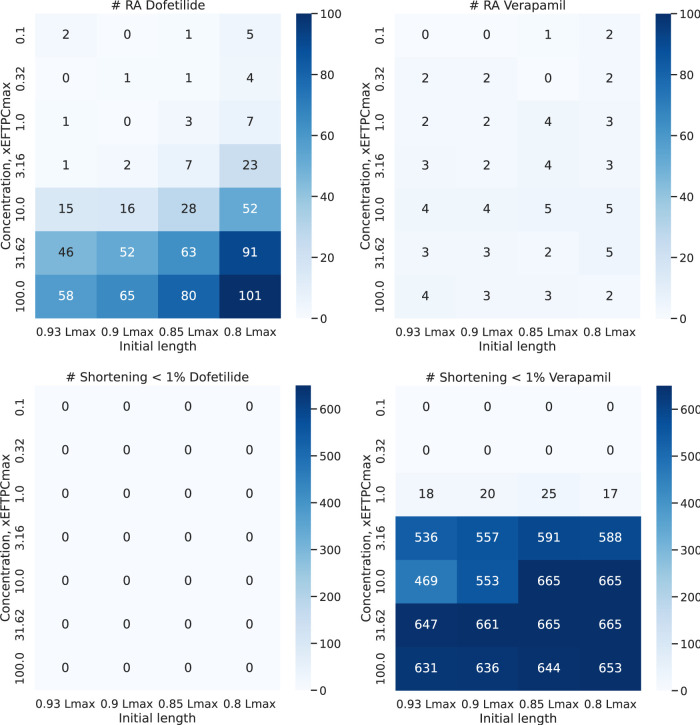
Abnormal events under Dofetilide and Verapamil. Heatmap shows a number of models with repolarization abnormalities (top line) and failed contraction (bottom line) in the final population of 674 models during contractions at different initial cell lengths from 0.93 to 0.80*L*_max_ with increasing drug concentration. The vertical axis plots the concentration of the compound (from 1 to 100xEFTPCmax), and the horizontal axis plots the initial cell length.

To assess the risk of adverse events in our population of calibrated electro-mechanical models under drug exposure, we estimated the frequency of the following events in the models. We recorded the occurrence of electrical disturbances as repolarization abnormalities and mechanical disturbances as failed contractions with an amplitude of isotonic shortening of less than 1%. Figure 10 shows a heatmap of the frequency of adverse events in our population of models with increasing drug concentration. Dofetilide demonstrated an increasing number of models with repolarization anomalies in the population both with increasing the concentration of the compound and with decreasing the initial sarcomere length (up to 101 models with repolarization anomalies at 100xEFTPCmax and initial length equal to 0.80*L*_max_, which is 15% of the model population). This result is consistent with experimental data characterizing Dofetilide as a drug with a high risk of arrhythmia. At the same time, Dofetilide shows no inotropic effect, and even high concentrations of Dofetilide do not affect the amplitude of model contractions in the population compared to the baseline.

For Verapamil, opposite effects are observed. Verapamil does not show an impressive electrophysiological effect: the number of models with repolarization abnormalities does not change significantly with concentration and initial sarcomere length. The model, therefore, predicts the drug as having a low risk of arrhythmia. At the same time, starting from a concentration of 3 times EFTPCmax and higher, Verapamil crucially reduces contraction ability in the vast majority of models from our population at any preload. This predicts the drug to be potentially dangerous in terms of contraction failure.

## 4 Discussion

### 4.1 Mechanical tests for calibration and verification of electro-mechanical cell models

In this study, we have built a new population of cell models of the electro-mechanical activity in human ventricular cardiomyocytes. First, the population we developed was calibrated and evaluated using experimental data on the biomarkers of cell electrophysiology and several tests for the effects of ionic current modulation on the AP shape and duration. Then, to the best of our knowledge, this is the first population that was also calibrated and evaluated using a combination of biomarkers of mechanical activity in human ventricular cells and several tests for the effects of mechanical interventions on cellular activity.

In the first step of the algorithm that we developed to build a population of calibrated models, we applied a HM approach to calibrating the models againts experimental data on the AP and *Ca* ^2+^ transient characteristics in isolated human ventricular cardiomyocytes (Table 1). The HM approach allows one to account explicitly for uncertainty and variability in observations and to define feasible regions of the model parameters that produce results within the experimental variability (Coveney and Clayton, 2018). In contrast to the studies by B. Rodriguez’s group, we took a more subtle approach of not rejecting models from the sampled population itself, but rather of restricting the space of input parameters using Gaussian process regression models and the HM method. This approach allowed us to remove the parameter ranges that gave rise to models with implausible (according to experimental biomarker values) behaviour. The result was a significant reduction in the initial input parameter space, ensuring an acceptance rate of 0.8 for models sampled from the reduced non-implausible parameter space. In other words, on average, 8 out of 10 models with parameters randomly sampled from the non-implausible space will show correct behaviour. This rate is much higher than the rate of 0.4 shown in Passini et al. (2017).

Previous modeling studies focused on the identification of the local input parameter distribution to reproduce variability in a particular experimental output set (Coveney and Clayton, 2018). In contrast, we have tested a wide range of parameter values to reproduce the wide variability in cardiac cellular electrophysiology and *Ca* ^2+^ handling as recorded in different experimental studies on isolated cardiomyocytes with comparable experimental conditions, while taking into account the uncertainty in these data. The approach we developed can be considered as an extension of the population of models approach (Britton et al., 2013; Muszkiewicz et al., 2016; Passini et al., 2017) where by the distributions of certain parameters were derived by calibration against AP recordings using acceptance/rejection criteria.

A different approach was suggested by Pathmanathan et al. (Pathmanathan et al., 2020) to develop populations of models based on the input parameter uncertainty assessed in experimental studies. In this work, the authors used a simplified model of the electrophysiology of the canine heart cell. The authors estimated the variability of a majority of input parameters based on experimental data on their distributions and used the Monte Carlo method to generate vectors in the input parameter space and then calculate electrophysiological models and estimate biomarkers.

In contrast to this work, we used an electro-mechanical mathematical model of the human cardiac cell for which there were no data on the uncertainty of the chosen input parameters. Therefore, we took advantage of available experimental data on several output biomarkers: the mean and uncertainty (variance) of the distributions of AP characteristics, min-max values of *Ca* ^2+^ transient characteristics and active force signals. Based on the experimental distributions for the model output biomarkers, we solved a kind of inverse problem, i.e., to find a multi-dimensional distribution of the input parameters that provides a distribution of output biomarkers within the experimental range. Similar to the Pathmanathan and Gray approach, each new model sampled from the non-implausible parameter space has to be tested in benchmark experiments for normality/abnormality in terms of biomarker values and the time course of AP, *Ca* ^2+^ transient and force/length twitch during contractions under different initial conditions.

The initial set of electrically calibrated models consisted of approximately 80% (769 out of 1,000) randomly selected models from a non-implausible parametric space defined by the HM algorithm in the last iterative step. The remaining 20% of models were rejected because they did not meet the calibration criteria for AP and *Ca* ^2+^ transients and/or had repolarization anomalies (RA, Supplementary Figure S4). Note that these 20% parameter sets were selected from the parametric space predicted as non-implausible by the Bayesian regression model (emulator) trained to predict the outputs of the original ODE model (simulator). The high number of rejected simulator models (compare the numbers of rejected emulators and rejected simulators in Figures 1B,C)) points to another facet of the uncertainty in the models. In paticular, it does not allow a regression model to predict the outputs of an ODE cell model with ≈100% high accuracy. This fact requires further analysis in order to improve the methods for generating populations of models.

Then, the initial population of electrically calibrated models was further re-calibrated using biomarkers of mechanical activity in human ventricular cardiomyocytes and various mechanical tests (Table 1; Figure 2). Such mechanical calibration of models was performed for the first time. We found more than 10% of electrically calibrated models (79 out of 769) that did not meet mechanical calibration criteria (Figure 2). The majority of the models (56 out of 79) rejected in this step showed repolarization anomalies during the excitation-contraction cycle at a reduced initial cell length compared to the reference length utilized to calculate cell activity for electrical calibration (Figure 3). The initial cell length (pre-stretch) in the intact heart depends on the mechanical preload imposed on the cells, which may vary between and within ventricles depending on the diastolic pressure in the ventricular chambers (Sengupta et al., 2006; Ashikaga et al., 2007). Such cardiomyocyte length variation in the physiological diapason should not induce ventricular excitation abnormalities in the normal heart, thus we rejected the models showing abnormal sensitivity to the preload.

A further test for the mechanical calibration of the models was also associated with the response to variation in initial cell length characterized by the ‘length - force’ (L-F) dependence. The isometric L-F relationship for an isolated cardiac preparation is commonly considered as an equivalent of the Frank–Starling law of the heart characterizing the ability of the intact heart to produce higher stroke volumes with increasing diastolic preload (Sequeira and van der Velden, 2015). In agreement with experimental data (Vahl et al., 1997; Holubarsch et al., 1998) (Table 1), the L-F dependence was assumed to be monotonous and rising with increasing the length, and not to fall down to near zero even at low (but permissible) initial cell lengths in the physiological diapason. Only two models that passed the preceding calibration steps did not meet the criteria for the L-F curve and were rejected (Figure 4). Such qualitative robustness of the L-F dependence in a wide range of model parameters indicates that our electro-mechanical cell model adequately reproduces this fundamental property of the myocardium.

Another fundamental characteristic of myocardial mechanics is the ‘force - velocity’ (F-V) dependence, which shows an increase in the shortening velocity of the contracting muscle with decreasing mechanical load (afterload). Three more models were additionally rejected as revealing repolarization anomalies during afterloaded contractions. Moreover, these three models displayed a non-monotonic F-V relationship, in contrast to that conventionally registered in the myocardium of all mammals (Figure 5). It should be noted that the three models did not show repolarization anomalies in isometric twitches at any initial lengths we tested. At the same time, faster and deeper sarcomere shortening during afterloaded contractions at low afterloads caused greater *Ca* ^2+^ release from TnC that was able to induce EADs in the models.

Finally, the 674 electrically and mechanically calibrated models, that passed all the tests we used for model calibration yielded action potential, *Ca* ^2+^ transient, and active tension that morphology and physiologically essential features (including time to peak, amplitude, recovery constant, and duration) are in agreement with experiments for human ventricular preparations. Similar to the human electro-mechanical cell model developed recently by Margara and co-authors (Margara et al., 2021), our calibrated models correctly predict the responses of human myocardial samples to modulations in the activity of several essential ionic currents affecting the AP characteristics.

It should be noted that variations in the mechanical conditions of cell excitation and contraction affect the AP characteristics and can induce not only contraction but also excitation abnormalities due to mechano-electric feedback mechanisms, which are taken into account in our electro-mechanical cell model. In our recent article (Balakina-Vikulova et al., 2020), we carefully analyzed the effects of mechano-electric coupling in human ventricular cells when evaluating the TP + M model. In the current study, the rejected model samples, that failed to pass the functional mechanical tests, predict that, under certain conditions, intra- and inter-cellular mechano-electric couplings can increase the vulnerability of arrhythmia in the intact myocardium during cardiac cycles.

### 4.2 Non-implausible parametric space and prediction of excitation abnormalities in sampled models

The non-implausible parameter space was essentially reduced as compared with the initial hypercube used to sample parameters for HM approach (Figure 6). Specifically, the low border for permissive values was distant from zero for conductivity of essential currents (*g*_*CaL*_, *P*_*NaK*_, *V*_*maxup*_, and *K*_*NaCa*_) strongly affecting characteristics of AP and *Ca* ^2+^ transient in normal cells.

No correlation was observed between the model parameters except the two parameters (*g*_*CaL*_ and *P*_*NaK*_) of the ionic currents defining *Ca* ^2+^ levels in the cell. In our previous simulation studies (Sulman et al., 2008; Katsnelson et al., 2011; Solovyova et al., 2016; Kursanov et al., 2023), we showed that an imbalance between the two currents particularly due to *I*_*NaK*_ inhibition may cause *Ca* ^2+^ overload followed by excitation disturbances (EADs, DADs, premature APs and extrasystoles) in cardiomyocytes and myocardial tissue. Furthermore, our models predicted, and subsequent wet experiments with ouabain confirmed, that the mechanical conditions of myocardial contraction (preload and afterload, and the mechanical interactions between cardiomyocytes and/or between cardiomyocytes and fibroblasts) may contribute to the pro-arrhythmic effect of *I*_*NaK*_ inhibition (Sulman et al., 2008; Katsnelson et al., 2011; Solovyova et al., 2016; Kursanov et al., 2023). The new data we revealed in the current study from the population of models suggest that the arrhythmogenic threshold for *I*_*NaK*_ inhibition strongly depends on the activity of *I*_*CaL*_ as well.

However, the varying parameters in the accepted models have distributions with rather high variability within the range of population sampling. Moreover, the analysis of parameter distribution for each of the 11 varying input parameters in the finally accepted models and models rejected during consequent electrophysiology calibration and mechanical tests showed no significant difference between the groups (Supplementary Figures S6, S7) except the only maximum velocity *V*_*maxup*_ of SERCA pump extruding cytosolic *Ca* ^2+^ into the SR during contractile cycles in the cells. The mean value of *V*_*maxup*_ was significantly higher in the rejected models compared with accepted models (Figure 8). At the same time the intervals of *V*_*maxup*_ value partially overlap in the accepted and rejected models, not allowing to rigidly separate the permissive and non-permissive values.

It is easy to demonstrate that intersection of projections of a multi-dimensional parameter space onto the low-dimensional subspaces no necessarily reflects the fact of inseparability between two multi-dimensional sets. There could be an explicit or implicit nonlinear transformation of the parameter space which separates the sets. In the case of the 11-dimensional space we analysed, we have not been able to find such a transformation that distinguishes the parameter sets of the accepted and rejected models.

Moreover, we think we should not assume a pure (deterministic/mechanistic) separability between the parameter subspaces for accepted and rejected models in the non-implausible parameter space we identified. Taking into account a high nonlinearity of the model solution on the parameter values, we could assume the possibility of non-physiological or unstable solutions in the deterministic ODE system at any given parameter vector, and consider a problem of predicting the model rejection.

We applied machine learning algorithms to assess the contribution of the 11 model parameters (input features) to the logistic regression score (see Supplementary Figure S9 in the Supplementary Materials). This analysis revealed the *V*_*maxup*_ parameter having the most essential contribution to the prediction of the score of model rejection. We also showed an increasing frequency of the rejected models with the *V*_*maxup*_ scaling factor (Supplementary Figure S8). This led us to develop a one-parametric logistic regression that showed the predictive power of the parameter in classifying models into the accepted and rejected sub-populations with high accuracy. The analysis revealed a threshold of *V*_*maxup*_ with a scaling factor of 1.2 that statistically separated the models.

Based the analysis, we have to stress that there is a non-zero chance to sample a model from the non-implausible parametric space that exhibits excitation abnormalities. This follows from the inherent uncertainty in the input and output parameters underlying the nature of biological subjects and the population construction algorithm we used. However, we have to point out that we analyzed the parameters of models from the non-implausible parametric space that was initially calibrated against the AP and *Ca* ^2+^ transient biomarkers. And this space was essentially reduced as compared with the wide space we used for the first model sampling ensuring a high acceptance rate of normal models. Despite the probabilistic nature of our predictions on the distribution of non-implausible parameters, our population of models that were initially calibrated according to the AP and *Ca* ^2+^ transient biomarkers showed a great fraction of models that passed all mechanical tests (674 out of 769, or 88%) we performed suggesting its normality. Moreover, in the range of *V*_*maxup*_ with a scaling factor from 0.3 to 1.2 the fraction of stable models was more than 95% (Supplementary Figure S8), and the fraction is about 70% if *V*_*maxup*_ is higher than 1.2. These results show the applicability of our approach for improving the model selection process with statistically expected predictions. At the same time, our analysis suggests that any model from the non-implausible parameter space we found should be further tested for normality before being used in *silico* studies of the effects of interventions.

### 4.3 Drug testing

In drug development, cardiac safety testing has been focused on life-threatening pro-arrhythmic events, especially Torsade de Pointes (TdP), a rare ventricular tachyarrhythmia that can lead to sudden death (Gintant et al., 2016). The recently launched Comprehensive *In Vitro* Proarrhythmia Assay (CiPA) initiative is aimed at developing a new paradigm integrating a set of predominantly nonclinical assays and methods for assessing the risk of TdP (Colatsky et al., 2016). Also, great efforts have been made to develop and use *in silico* electrophysiological and electro-mechanical cardiomyocyte models (Chang et al., 2017; Passini et al., 2017; 2019; Li et al., 2019c; Tomek et al., 2019; Margara et al., 2021).

Drug effects are typically incorporated in cell models using IC50 and Hill coefficient data (Passini et al., 2017), by means of simple pore-block models of drug action. The effects of ionic current modulation on the AP wave and force generation relative to control have been compared to experimental data available on human cardiomyocytes and myocardial objects and different metrics of excitation abnormalities in drug exposure simulations have been suggested for predicting the pro-arrhythmia risk of drugs and comparing it with pre-clinical and clinical data. As generally agreed in the CiPA community, *in silico* mechanistic models have a great potential for drug testing (Colatsky et al., 2016; Li et al., 2019a).

To validate our population of accepted electro-mechanical models of human cardiomyocytes, we also used two multichannel action reference compounds from the list of 28 so-called “calibration drugs” suggested for lab-specific calibration and validation of CiPA models for pro-arrhythmic risk prediction (Colatsky et al., 2016; Li et al., 2019a). We selected Dofetilide and Verapamil as representative compounds classified into the three “High”, and “No or Very low” TdP risk groups according to available data and expert clinical opinion (Li et al., 2019a). We validated our *in silico* population of virtual cardiomyocytes using *in vitro* ion channel data for the drugs incorporated into each accepted model of our calibrated population. The model responses were compared with experimental and clinical data for the drug effects on electrophysiology and contractility in human subjects (see the reference list in (Margara et al., 2021)). In consistency with the clinically categorized pro-arrhythmic profiles of the drugs, their specific effects on the ionic currents differently affected the cellular electrical and contractile activity in the models. In accordance with experimental data (Gibson et al., 2014; Page et al., 2016; Britton et al., 2017), Dofetilide prolonged the AP duration in the models at low and moderate concentrations (Figure 9). With increasing the concentrations further, the drug, which is known to be clearly associated with TdP risk, caused an increased incidence of repolarization abnormalities in the models (Figure 9; Figure 10). In addition, the probability of adverse events under Dofetilide-induced modulation of the ionic currents was shown to be significantly dependent on the mechanical conditions of excitation, increasing with decreasing mechanical preload on the virtual cells. For example, under a Dofetilide concentration of 10xEFTPCmax, the frequency of repolarization abnormalities was more than three times higher at an initial length of 0.80*L*_max_ versus 0.93*L*_max_ (Figure 10). In contrast, Verapamil, classified as a safe drug with no risk of TdP, had little effect on AP duration and showed a low frequency of repolarization abnormalities in the models even at a drug concentration as high as 100xEFTPCmax. Moreover, the incidence of adverse events was virtually independent of the Verapamil concentrations we tested and of the mechanical conditions we varied (Figure 9).

Contrary to the effects on the electrical activity in virtual cardiomyocytes, Dofetilide had almost no effect on the mechanical activity at any concentrations. No failed contraction (assumed as less than 1% shortening in the afterloaded isotonic twitch) was observed at any Dofetilide concentration for any initial cell length we tested. In contrast, Verapamil was shown to induce an abrupt negative inotropic effect at concentrations above 3xEFTPCmax, with the majority of models ceasing to contract despite a near-normal excitation profile. In this case, the frequency of mechanical abnormalities showed no specific dependence on the initial cell length, demonstrating an “all or nothing” effect independent of the external mechanical condition.

The drug testing results confirm that our population of calibrated electro-mechanical models is potentially appropriate for drug testing according to the general principles declared in the White Paper of the CiPA community for creating, validating and establishing the acceptability of *in silico* models for pro-arrhythmic risk prediction (Li et al., 2019a; b). The next steps of population validation will require *in lab* testing of the whole set of the selected 28 “calibrating” and “validating” compounds, and development of *in lab* metrics for assessing the risk of excitation abnormalities and contractile dysfunction consistent with available experimental and clinical data and predictive of the category of TdP risk and other adverse events as agreed in the community.

## 5 Strengths and limitations

The key accomplishment of our paper is that we have clearly demonstrated that the mechanical conditions during cardiomyocyte excitation-contraction cycles are essential factors affecting the myocardial performance *in silico* cardiac models. Changes in the external and internal mechanical conditions for the myocardial activity, such as preload (stretching at diastolic pressure) and afterload (aortic resistance and systolic pressure), the mechanical environment of each cardiomyocyte in the tissue and their activation sequence, occur to a greater or lesser extent in every cardiac cycle of our daily lives. This determines the dynamic processes of cellular excitation and contraction, which are closely coupled with each other by feedforward and feedback links. We have showed that at certain combinations of cellular properties of ionic currents and *Ca* ^2+^ handling, a change in the mechanical conditions may cause excitation abnormalities, which are not revealed by other mechanical tests. We discovered that a number of models carefully calibrated using various electrophysiology tests and demonstrating normal AP and *Ca* ^2+^ transient signals with acceptable biomarkers may reveal anomalies in the mechanical tests. Thus, our results highlight the importance of mechanical testing for the generation, calibration, verification, and further usage of *in silico* models for different tasks of basic science and applications. The key message of our study is that cellular mechanics need to be taken into account even if one consider only abnormalities in electrical activity in response to an intervention involving predominantly ionic mechanisms!

However, there is another cellular mechanism of transfer mechanical of impacts on AP generation in cardiomyocytes that has not been in the focus of our simulations. The activity of stretch-activated and mechanosensitive channels could explain some phenomena related to acute or chronic changes in the mechanical environment of cardiomyocyte contraction (Quinn and Kohl, 2021). Previously, we discussed the use of stretch-activated channels in simulations by the TP + M model (Balakina-Vikulova et al., 2020). We argued that, according to experimental data, stretch-activated channels have a role to play more likely in slow responses to mechanical changes than in our tests dealing with immediate responses in cardiomyocyte excitation to changes in the mechanical loading during one pre- and afterloaded contractions. The uncertainty and diversity of experimental data for the parameters responsible for the reversal potentials and conductance of stretch-activated channels complicate the correct insertion of these channels in the TP + M model. However, we plan to make further efforts to correctly implement the stretch-activated channels in the reference TP + M model, and then in the respective population so that they could be applied to the analysis of disturbances in the electromechanical activity of cardiomyocytes during slow force responses and prediction of effective scenarios aimed at correcting such disturbances.

Our population of models was generated assuming variability in parameters that define the activity of several transmembrane ionic currents and *Ca* ^2+^ release from and uptake into the SR. These parameters affect significantly the AP and *Ca* ^2+^ transient shape and physiologically important characteristics in the cells. Model calibration revealed several relationships between the parameters, that coordinate ionic levels and dynamics in normal cells predicted from unexpected disturbances. We found great variability in the selected parameters defining the variability in the cellular output biomarkers in the experimentally permissive range. We showed that the parameters of intracellular *Ca* ^2+^ handling, especially the maximum velocity of the SERCA *Ca* ^2+^ pump performing *Ca* ^2+^ uptake from the cytosol into the SR, affect the excitation profile, and its increase over a threshold permissive level may cause excitation anomalies.

In this study, we did not vary the intrinsic parameters of the mechanical activity (such as the velocity of cross-bridge cycling, cooperativity of *Ca* ^2+^ activation of myofilaments and so on) between the models. However, these parameters are known to vary between cells and cycle-to-cycle depending on the conditions (Cordeiro et al., 2004; Stelzer et al., 2008; Cazorla and Lacampagne, 2011). Particularly, in our recent study using an electro-mechanical model of cardiomyocyte and neural networks (Parikh et al., 2022), we predicted that these model parameters are strongly affected by the drug Omecamtiv Mecarbil underlying its inotropic action on myocardium objects.

Furthermore, we did not classify our population of cell models into groups reflecting the regional heterogeneity in the properties of cardiomyocytes in different regions and layers of the ventricular myocardial wall. There are available experimental data on the cellular transmural (endo-, mid-, and epicardial) heterogeneity across the wall and longitudinal (from apex to base) heterogeneity with distinctive cardiomyocyte properties (Janse et al., 2012; Boukens et al., 2015; Solovyova et al., 2016). This issue was addressed in several simulation studies by us (Markhasin et al., 2012; Solovyova et al., 2014; Khokhlova et al., 2017; 2018) and other authors (Seemann et al., 2003; Bondarenko and Rasmusson, 2010; Maoz et al., 2014), including models of human cardiomyocytes starting from the original TNNP and ORd models of cell electrophysiology (O’Hara et al., 2011; Christophe, 2013; Vandersickel et al., 2016; Kojima et al., 2020) to their recent updates (Tomek et al., 2019; Margara et al., 2021). In recent articles from our group (Khokhlova et al., 2020), the distinctions in the intrinsic parameters of contractile activity were shown to be essential for reproducing the distinctions we revealed in isolated cardiomyocyte experiments in response to changes in the mechanical conditions of contraction. We are going to address the cellular heterogeneity using our population of models to reveal the mechanisms underlying the different responses of cardiomyocytes from different ventricular regions to various electrical and mechanical interventions in norm and pathology.

Experimental and theoretical studies have shown that the interaction of heterogeneous cardiac muscle preparations can significantly alter the characteristics of contraction and AP in interacting samples (Solovyova et al., 2016; Vikulova et al., 2016). The presence of an excitation delay between different regions of the ventricular wall causes some cardiomyocytes to activate earlier and stretch the cardiomyocytes that are not yet activated, changing the mechanical environment for their contraction. Due to the mechanisms of mechano-electric feedback and electrotonic interaction between adjacent cardiomyocytes, the generation of AP is also altered compared to a single, uncoupled cardiomyocyte (Quinn and Kohl, 2021). Heterogeneous cells that are mechanically and electrically coupled in the normal or pathological myocardium could react differently to electrical or mechanical impacts compared to single cells. In particular, cells in the whole heart, which are under considerable electronic load from electrically-coupled neighbouring cells, should have a lower potential for abnormal focal excitation under variations in pre- and afterload. Our preliminary results obtained by using the continuous model of 1D heterogeneous cardiac muscle confirm this. We have shown that extra APs and delayed afterdepolarization occurring in a single cell become weaker (Vikulova et al., 2015) or disappear (not published) in a strand of electro-mechanically interacting cardiomyocytes. By way of summarising, it would be a great challenge to study the behaviour of electromechanical models of 1D, 2D and 3D samples of cardiac tissue consisting of communicating models obtained during the construction of populations, including the cellular transmural or pathological heterogeneity of cells.

## 6 Conclusion

We calibrated a large number of our *in lab* electro-mechanical models of human cardiomyocytes selected randomly from a parametric space using a great variety of experimental data available on the electrophysiology, *Ca* ^2+^ transient and contractile activity of isolated cells and myocardial preparations from human ventricular myocardium. For the first time, the models were calibrated using mechanical tests with different mechanical preloads and afterloads. One of the main results of our study is that the mechanical tests are necessary not only for the evaluation of the mechanical activity in the cells but for both *Ca* ^2+^ handling and electrical activity. For the first time, we revealed that cell electro-mechanical models, which were thoroughly calibrated and evaluated to simulate electrical activity and *Ca* ^2+^ transient in normal cardiomyocytes may demonstrate abnormal behaviour under mechanical tests.

Finally, we built a population of accepted models, which were calibrated in electrophysiological and mechanical tests and yielded normal waveforms of AP, *Ca* ^2+^ transient, force and length change during contractile cycles at different pre- and afterloads, with amplitude and time-dependent characteristics being representative of the variability in experimental observations in normal cells. The accepted models are robust to variation in mechanical conditions within the permissive range of parameter variations in terms of low pro-arrhythmia risk. At the same time, we revealed model parameters, particularly *V*_*maxup*_, which, when taken outside of the permissive range strongly increase the probability for excitation abnormalities in the models, especially under a change in the mechanical conditions.

The population of models was verified using “calibrating” drugs with a pre-described action on the ionic currents and previously categorized into the groups of either High or Low risk of TdP. Our models adequately reproduced the effects of Dofetilide and Verapamil on the cell AP waveform and contraction. The models predicted a high risk of excitation abnormalities under Dofetilide and a low risk of Verapamil. In contrast, Verapamil showed an essential negative effect on contractility and contraction disappearance at high concentrations of the drug. Our results predict that sensitivity to drugs may also be mechano-dependent, increasing vulnerability to arrhythmia induction under specific mechanical conditions.

In conclusion, we have created a population of electro-mechanical models of normal human cardiomyocytes that can be used for various basic science studies and applications. We have demonstrated that mechanical tests are essential at each stage of *in silico* model generation, calibration, verification, and further use.

## Data Availability

The raw data supporting the conclusion of this article will be made available by the authors, without undue reservation.
