# Supplementary material for: The importance of mechanical conditions in the testing of excitation abnormalities in a population of electro-mechanical models of human ventricular cardiomyocytes
Source: Front Physiol. 2023 Jun 8;14:1187956. doi: 10.3389/fphys.2023.1187956 (PMC10285544; doi:10.3389/fphys.2023.1187956)
Supplement: Supplementary file 1 [file DataSheet1.PDF]

Supplementary Material

SUPPLEMENTARY FIGURES

Figure S1.Combined computational pipeline scheme

Figure S2. Concentration-response relationship for a block of ion channels by Dofetilide

Figure S3. Concentration-response relationship for a block of ion channels by Verapamil

Figure S4. Examples of models rejected during the History Matching.

Figure S5. 'Length – force' relationship in the population

Figure S6. Scatter plot of the input parameters of the population

Figure S7. Scaling factors of input parameters in accepted and rejected models. Wasserstein distances between scale factor distributions

Figure S8. Increasing ratio of the rejected models with increasing the V_{maxup} scaling factor

Figure S9. SHAP plot for the Logistic regression mode

Figure S10. Scatter plot of output biomarker values in the population

Figure S11. Changes in AP in the model sampled from the final population under modulations of ionic currents

REFERENCES

Holubarsch, C., Lüdemann, J., Wiessner, S., Ruf, T., Schulte-Baukloh, H., Schmidt-Schweda, S., et al. (1998). Shortening versus isometric contractions in isolated human failing and non-failing left ventricular myocardium: dependency of external work and force on muscle length, heart rate and inotropic stimulation. *Cardiovasc. Res.* 37, 46–57. doi:10.1016/S0008-6363(97)00215-0

Kramer, J., Obejero-Paz, C. A., Myatt, G., Kuryshev, Y. A., Bruening-Wright, A., Verducci, J. S., et al. (2013). MICE models: Superior to the HERG model in predicting torsade de pointes. *Sci. Rep.* 3. doi:10.1038/srep02100

Passini, E., Britton, O. J., Lu, H. R., Rohrbacher, J., Hermans, A. N., Gallacher, D. J., et al. (2017). Human in silico drug trials demonstrate higher accuracy than animal models in predicting clinical pro-arrhythmic cardiotoxicity. *Front. Physiol.* 8, 668. doi:10.3389/fphys.2017.00668

Vahl, C. F., Timek, T., Bonz, A., Kochsiek, N., Fuchs, H., Schaffer, L., et al. (1997). Myocardial length-force relationship in end stage dilated cardiomyopathy and normal human myocardium: analysis of intact and skinned left ventricular trabeculae obtained during 11 heart transplantations. *Basic Res. Cardiol.* 92, 261–70. doi:10.1007/BF00788521

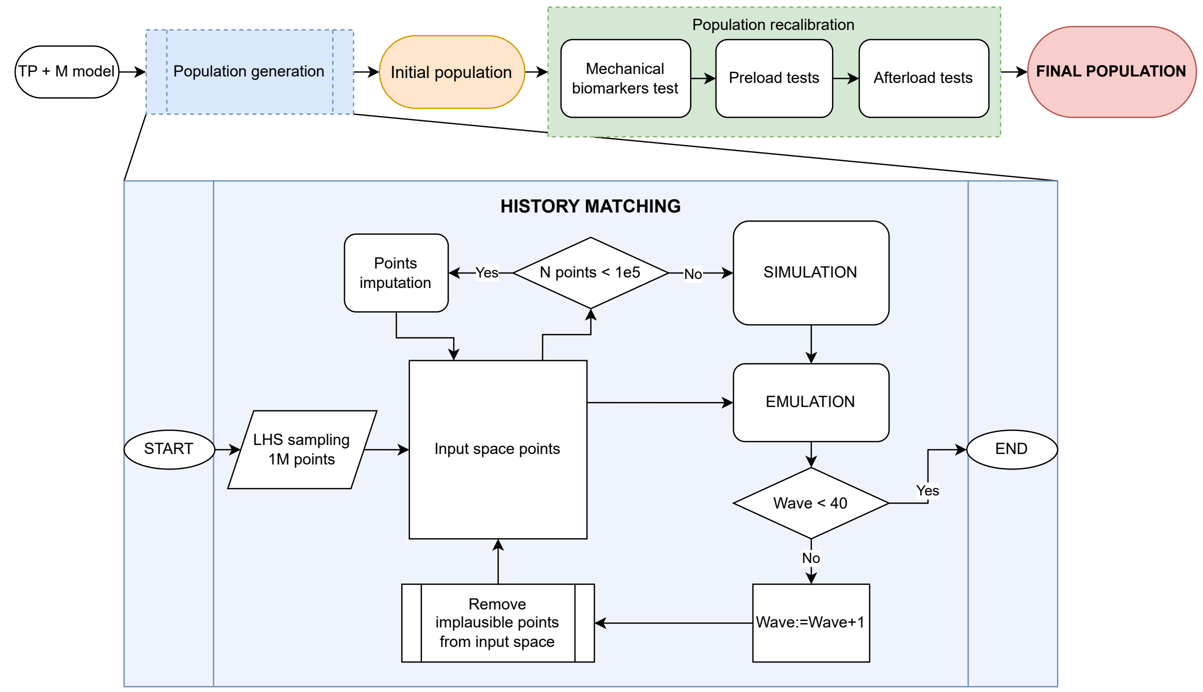

Figure S1. Combined computational pipeline scheme. The process of creating the **Final population** consists of the following steps: generation of the **Initial population** using the History Matching method and model calibration using electrophysiological and Ca^{2+} transient biomarkers followed by re-calibration using mechanical tests: rejection models with force biomarkers falling outside the experimental data; preload tests (isometric twitches at different initial lengths and consistency of 'length - force' curve with experimental data) and afterload tests (isotonic twitches and consistency of 'force - velocity' relationship with experimental data)

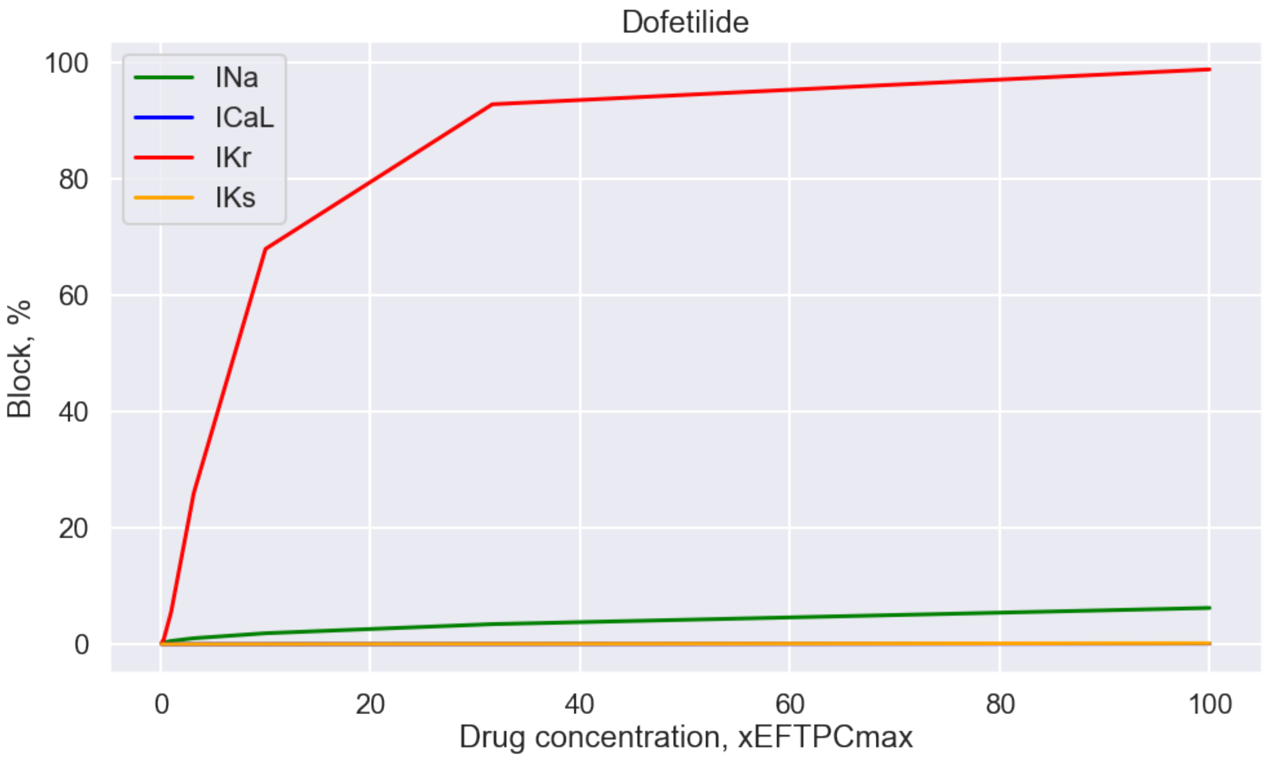

Figure S2. Concentration-response relationship for a block of ion channels by Dofetilide, computed with a simple pore-block model. IC50 and Hill coefficient are from (Passini et al., 2017)

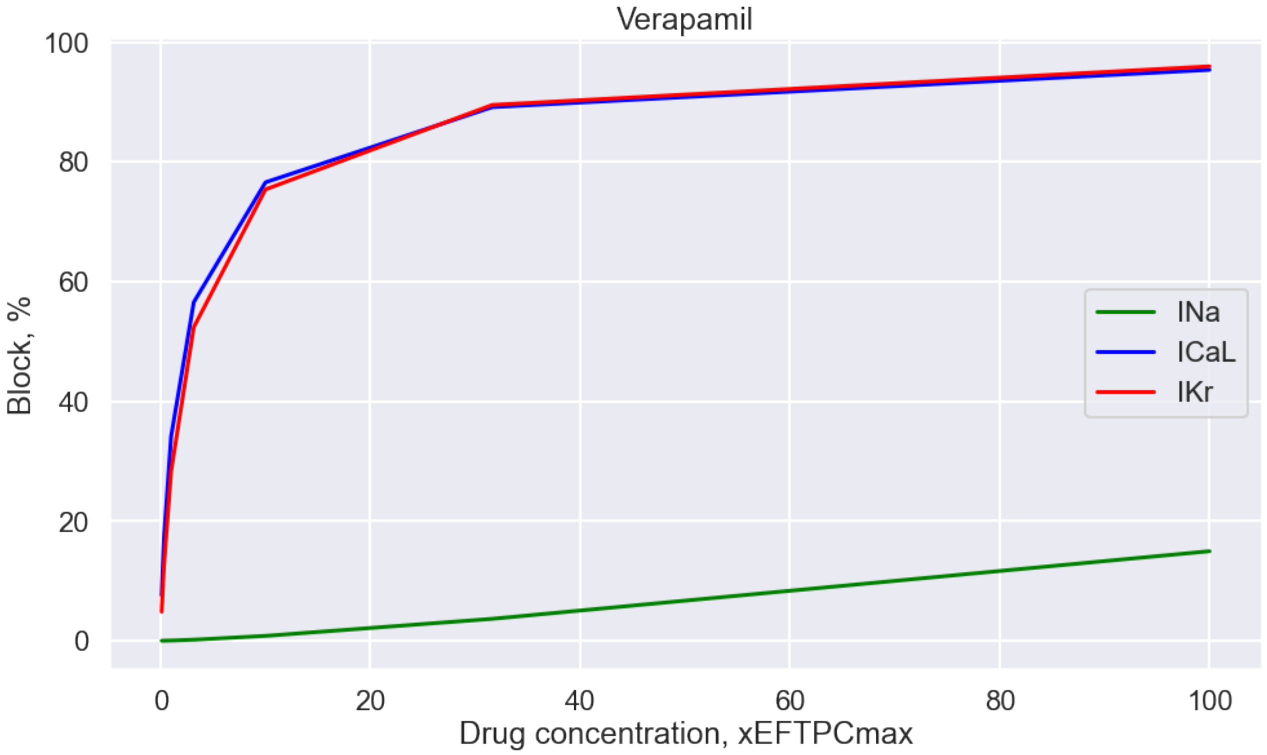

Figure S3. Concentration-response relationship for a block of ion channels by Verapamil, computed with a simple pore-block model. IC50 and Hill coefficient are from (Kramer et al., 2013)

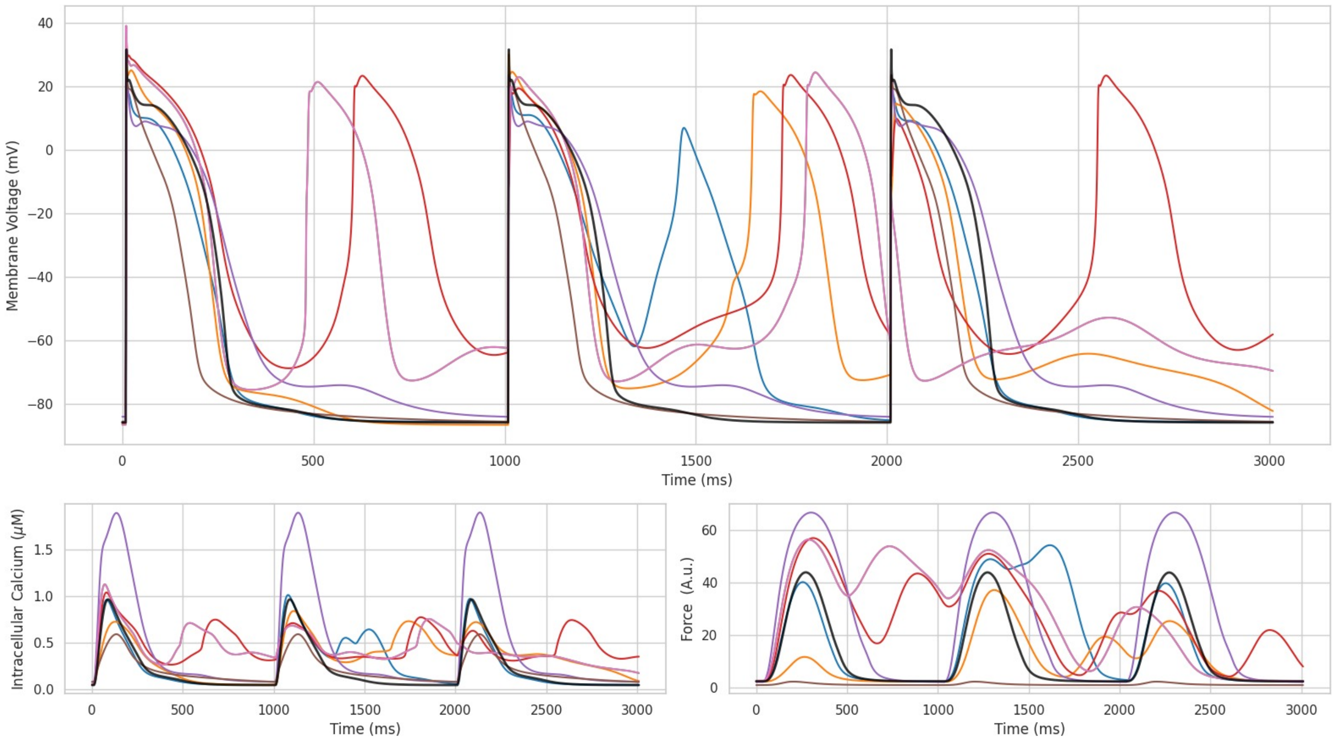

Figure S4. Examples of models that did not meet calibration criteria for AP and calcium transient and/or had repolarization abnormalities. Cardiomyocyte action potential, intracellular calcium, and active force generated by cardiomyocytes during steady-state isometric twitches.

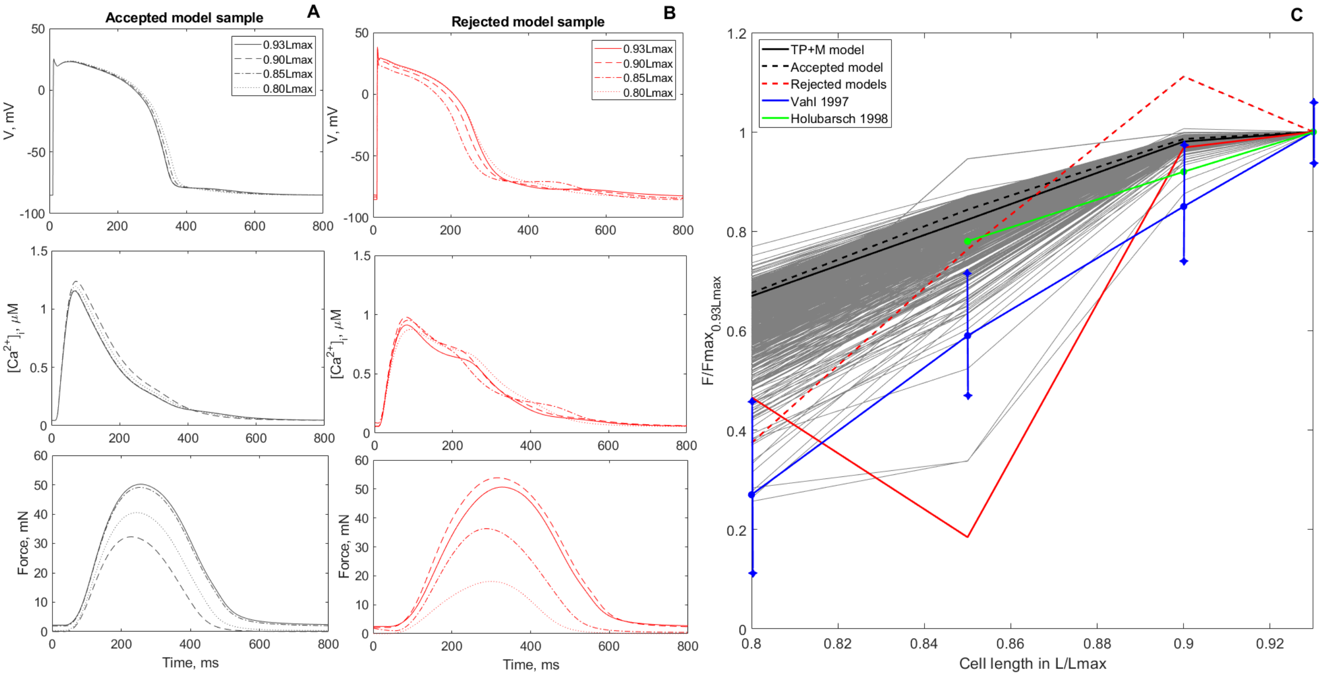

Figure S5. 'Length – force' relationship in the population of human ventricular cardiomyocyte electromechanical models. **A**: An example of an accepted model from a population. **B**: An example of a model that was excluded from the population. Panels show action potential (AP), Ca^{2+} transients ($[Ca^{2+}]_i$), and active force generated by the cardiomyocyte (Force) during steady-state isometric twitches at different initial lengths. **C**: 'Length – force' relationships for all models in the population that consisted of models without excitation abnormalities. The data obtained in experiments on human cardiac preparations are presented by the blue line Vahl et al. (1997) and the green line Holubarsch et al. (1998) The relationship for the TP+M is indicated by the black line. The accepted model sample is shown by the black dashed line. Rejected models are shown by red lines. The X-axis is the initial length of the cardiac virtual sample, normalized to L_{max} (the length at which the muscle develops maximal isometric force). The Y-axis is the isometric peak force value normalized to the isometric peak force in the corresponding model of the population when its initial length is equal to $93\%L_{max}$.

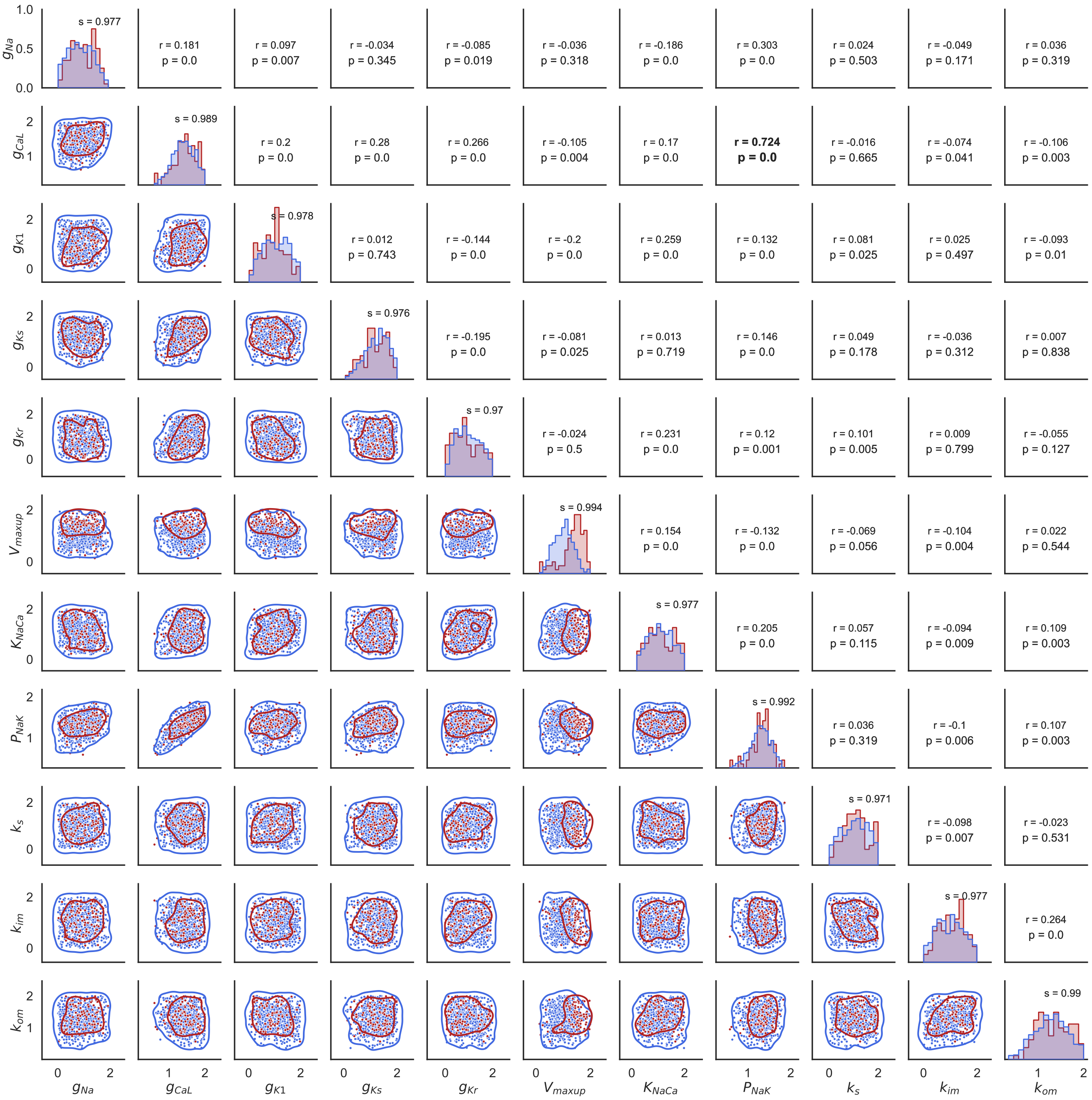

Figure S6. Scatter plot of the input parameters of the population of electro-mechanical models of human ventricular cardiomyocytes. Triangle bottom left: pairwise scatter plots of input parameters. Diagonal: histograms of the distribution of the values of each input parameter within the population. Upper right corner: Pearson's correlation coefficients and p-values, values of $r > 0.5$ are highlighted in bold. Blue for models from the final population and red for models rejected by biomarkers of force, with excitation anomalies at different initial lengths and discarded by 'length - force' and 'force - velocity' tests.

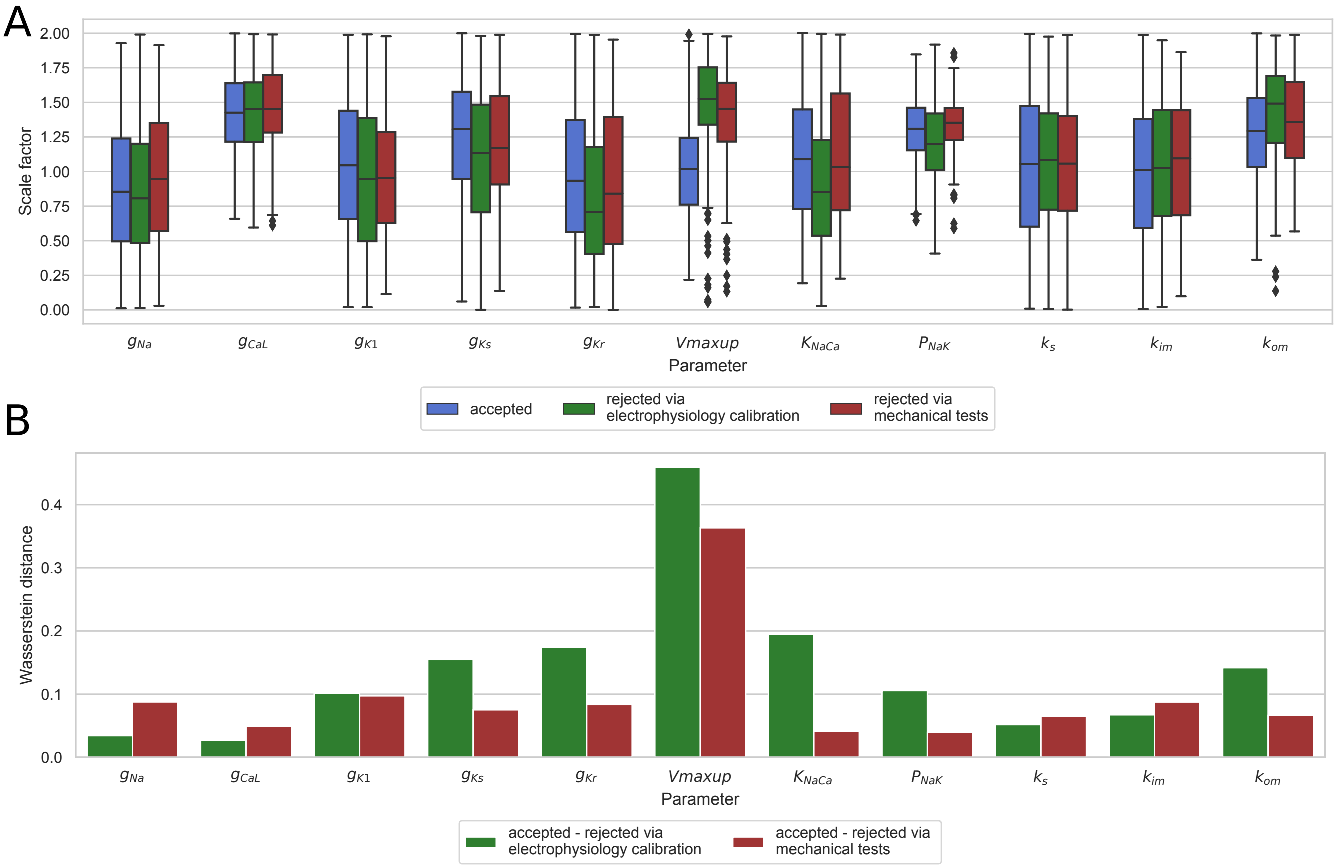

Figure S7. A. Scaling factors of input parameters in accepted (blue), rejected via electrophysiology calibration (green) and rejected via mechanical tests (red) models, shown as boxplot diagrams. The middle marker of the boxes shows the median, the box boundaries are the 25th and 75th percentiles, and the whiskers extend to the most extreme data points. B. Wasserstein distances (WD) between scale factor distributions: green - WD between accepted and rejected via electrophysiology calibration, red - WD between accepted and rejected via mechanical tests populations

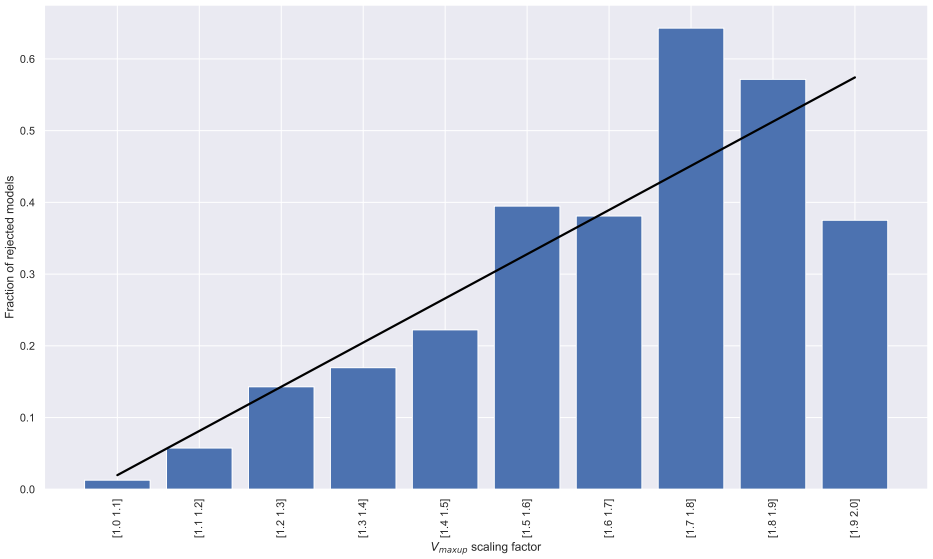

Figure S8. Increasing ratio of the rejected models with increasing the V_{maxup} scaling factor. The line indicates a linear trend. The vertical axis shows the fraction of rejected models, the horizontal axis shows the range of V_{maxup} scaling factors for each bar

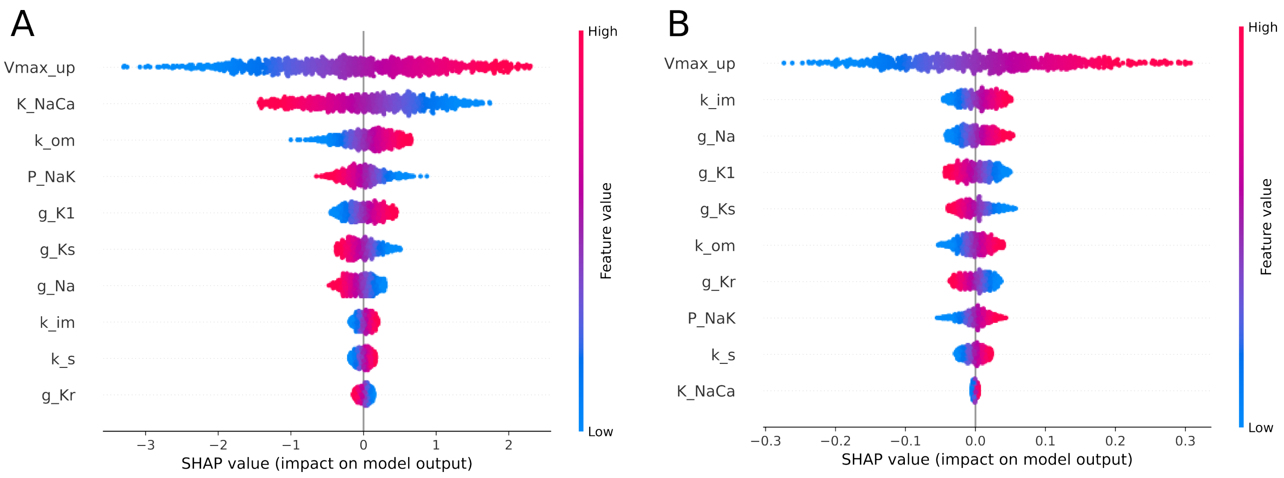

Figure S9. SHAP (SHapley Additive exPlanations) plot shows the feature importance for the Logistic regression model in classification via electrophysiology calibration (A) and mechanical tests (B) into rejected (1) or accepted (0) models. Each dot on the scatter represents an individual model in population, the width of the scatter indicates the parameter importance, and the colour indicates which direction of that feature value is predictive. g_{CaL} parameter was removed from the analysis due to its strong correlation with P_{NaK} (Pearson r > 0.7 for both cases)

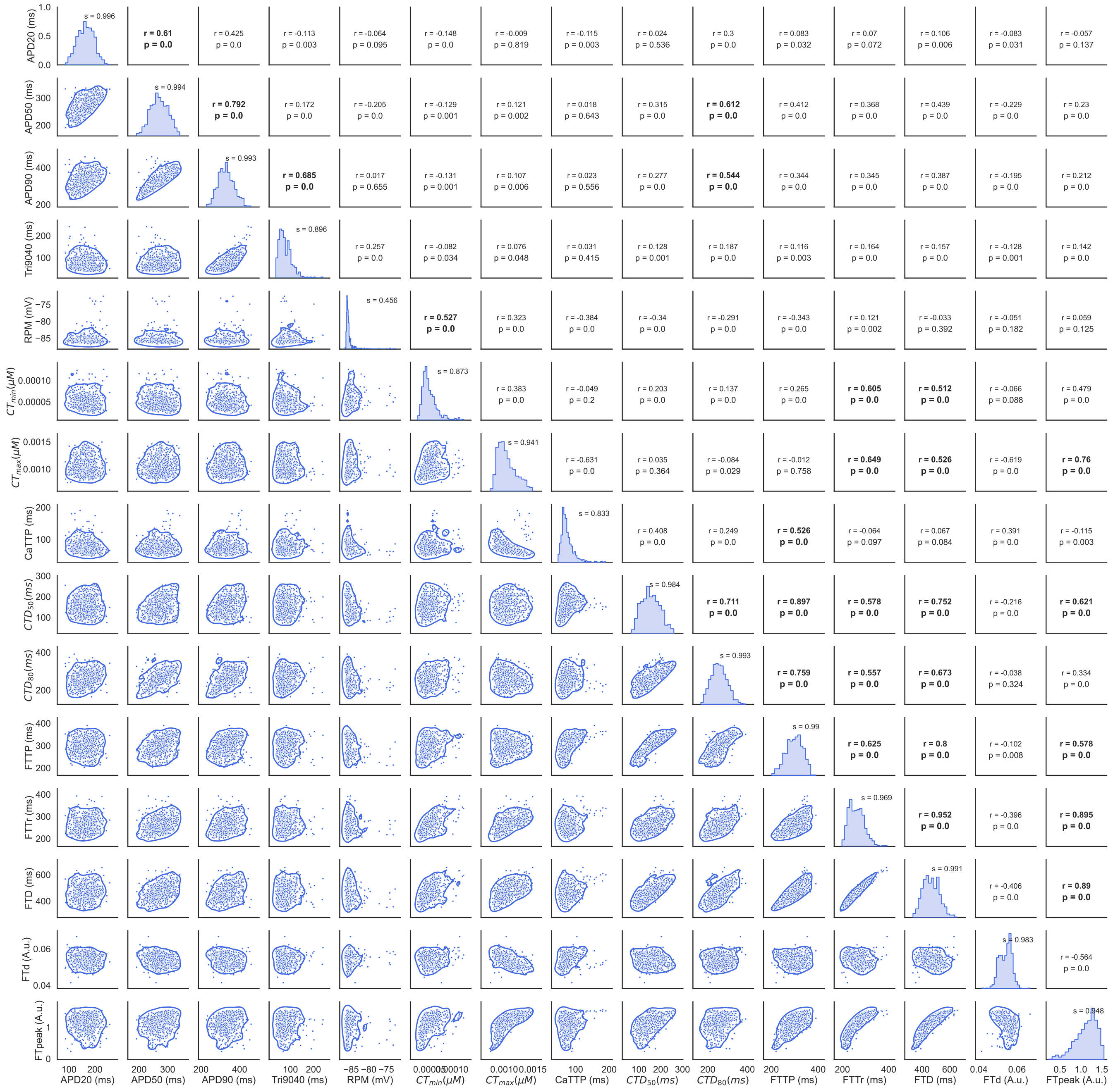

Figure S10. Scatter plot of output biomarker values in the population of human ventricular cardiomyocyte electro-mechanical models. Lower left corner: pairwise scatter plots of biomarkers, diagonal: distribution histograms of individual biomarker values in the population, upper right corner: values of Pearson's correlation coefficients and p-values, values of $r > 0.5$ highlighted in bold.

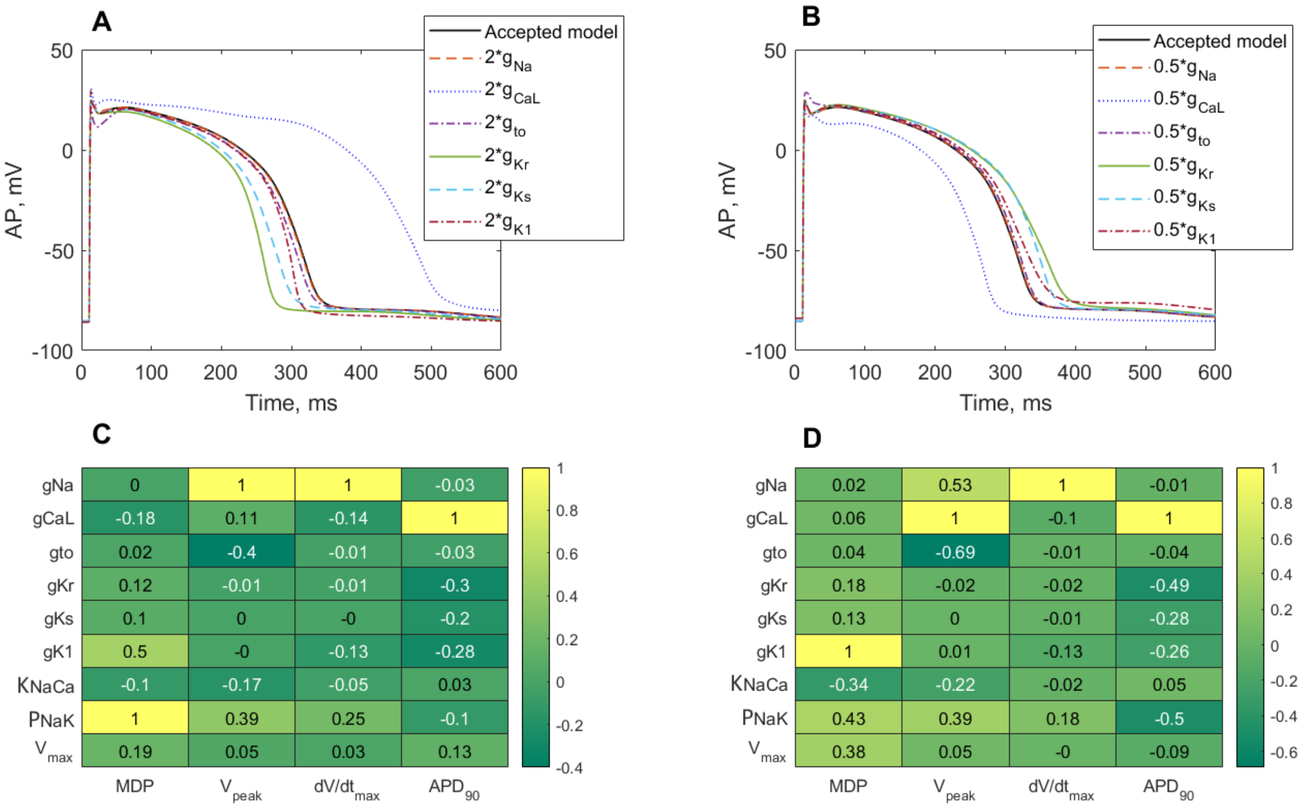

Figure S11. Changes in AP in the model sampled from the final population, with an increase of g_{Na}, g_{CaL}, g_{to}, g_{Kr}, g_{Ks}, g_{K1} by 2 (**A**) and 0.5 (**B**) fold. Relative sensitivities calculated from biomarker changes (maximum diastolic potential (RMP), peak voltage (V_{peak}), maximum upstroke velocity (dV/dt_{max}), and APD at 90% repolarization (APD_{90}), observed in the TP+M model (**C**) and in the accepted population model (**D**) when current conductance values were scaled between 0.5 and 2. Darker yellows/greens correspond to greater positive/negative relative sensitivity.
